# Cervical Lymph Nodes as a Selective Niche for *Brucella* during Oral Infections

**DOI:** 10.1371/journal.pone.0121790

**Published:** 2015-04-28

**Authors:** Kristine von Bargen, Aurélie Gagnaire, Vilma Arce-Gorvel, Béatrice de Bovis, Fannie Baudimont, Lionel Chasson, Mile Bosilkovski, Alexia Papadopoulos, Anna Martirosyan, Sandrine Henri, Jean-Louis Mège, Bernard Malissen, Jean-Pierre Gorvel

**Affiliations:** 1 Centre d'Immunologie de Marseille-Luminy (CIML), Aix-Marseille University, UM2, Marseille, France; 2 Institut National de la Santé et de la Recherche Médicale (INSERM), U1104, Marseille, France; 3 Centre National de la Recherche Scientifique (CNRS), UMR7280, Marseille, France; 4 University Clinic for Infectious Diseases and Febrile Conditions, Skopje, Republic of Macedonia; 5 Unité des Rickettsies, Aix-Marseille University, Centre National de la Recherche Scientifique (CNRS), UMR6020, Faculté de Médecine, Marseille, France

## Abstract

Cervical lymph nodes (CLN) are the first lymph nodes encountered by material taking the oral route. To study their role in orally acquired infections, we analyzed 307 patients of up to 14 years treated in the university clinic of Skopje, Macedonia, for brucellosis, a zoonotic bacterial disease frequently acquired by ingestion of contaminated dairy products. From these children, 36% had lymphadenopathy. Among orally infected children, lymphadenopathy with CLN being the only lymph nodes affected was significantly more frequent as compared to those infected by contact with animals (83% vs. 63%), suggesting a possible involvement of CLN during orally acquired human brucellosis. Using a murine model where bacteria are delivered into the oral cavity, we show that *Brucella* quickly and selectively colonize the CLN where they proliferate and persist over long periods of time for up to 50 days post-infection. A similar efficient though less specific drainage to CLN was found for *Brucella*, *Salmonella typhimurium* and fluorescent microspheres delivered by gavage, a pathway likely representing a mixed infection mode of intragastric and oral infection, suggesting a central pathway of drained material. Microspheres as well as bacteria drained to CLN predominately reside in cells expressing CD68 and no or low levels of CD11c. Even though no systemic response could be detected, *Brucella* induced a locally restricted inflammatory reaction with increased expression levels of interferon γ, interleukin (IL)-6, IL-12, granzyme B and a delayed induction of Nos2. Inflammation led to pronounced lymphadenopathy, infiltration of macrophages/monocytes expressing high levels of major histocompatibility complex II and to formation of epitheloid granulomas. Together, these results highlight the role of CLN in oral infections as both, an initial and efficient trap for bacterial invaders and as possible reservoir for chronic pathogens. They likewise cast a new light on the significance of oral routes for means of vaccination.

## Introduction

Everyday life confronts the immune system with permanent exposure to a multitude of different antigens. In order to react appropriately, constant sampling from the lumen of airways and gastrointestinal tract ensures capturing, transportation and presentation of those antigens in regional lymph nodes. In these organs, a tightly controlled balance of responses will result in induction of either tolerance or immunity. The oral cavity is the first site of contact with antigens taking the oral route. At the same time it is the habitat of microbial commensals as well as the point of entry of multiple particularly viral pathogens [1]. The oral cavity is drained by the cervical lymph nodes (CLN) [2] which also drain the eye [3], the central nervous system [4] and the nasal mucosa [5]. Similar to mesenteric lymph nodes (MLN) that have been shown to be central for mucosal oral tolerance induction [6,7], CLN are involved in mediating tolerance of oral and nasal mucosa [5,8].

A zoonotic bacterial disease that is frequently acquired by the oral route is caused by bacteria of the genus *Brucella*. These Gram-negative facultative intracellular pathogens infect a wide range of domestic and wild animals where they can cause long-lasting infections, often associated with sterility or abortion [9]. Human brucellosis or Malta fever is among the most common zoonosis worldwide caused by a bacterial pathogen [10]. Infection leads to a febrile disease of multiple unspecific symptoms including muscle pain, fatigue, hepato- and splenomegaly and tends to become chronic if it remains untreated. Infection occurs through aerosols, through wounds in contact with infected animals or by ingestion of contaminated dairy products [11]. *Brucella* species most relevant for human and livestock infections are *B*. *abortus* in cattle, *B*. *suis* in swine and *B*. *melitensis* in goats and sheep [9]. Even though mice do not represent the natural host for these species and eventually control *Brucella* infection even with large inoculums due to natural resistance, they are commonly used as animal model since they reproduce multiple aspects of the disease including bacterial initial replication and granuloma formation [12].

Here, we found that inflammation of the CLN occurs at higher incidence in children that have likely acquired brucellosis via ingestion compared to those probably infected by different routes. Since the most commonly used mouse models of *Brucella* infection bypass the tissues of the upper digestive tract drained by those lymph nodes, we established a mouse model of oral infection where bacteria are applied directly into the oral cavity. We found that CLN efficiently filter and trap bacteria as well as inert particles when administered either orally or by gavage, demonstrating CLN as master checkpoint of material taking the oral route. Using *Brucella* as a model for a chronic bacterial infection, we show that the bacteria quickly and selectively colonize the CLN after oral administration where they proliferate and persist over long periods of time, highlighting the role of CLN in infection as both, initial and efficient trap for bacterial invaders and as possible reservoir for chronic pathogens.

## Results

### CLN in human *Brucella* infections

To investigate the possible correlation of cervical lymphadenopathy in human brucellosis to infection by the oral route, we retrospectively analyzed a rather homogenous group of patients diagnosed with brucellosis comprising 317 children not older than 14 years old admitted to and treated in the University Clinic for Infectious Diseases and Febrile Conditions, Skopje, Republic of Macedonia during the period from January 1989 to December 2011. From these children, 167 had acquired disease by ingestion and 140 by contact with infected animals. For the remaining 10 children, route of infection was not known and they were therefore omitted from further analysis. From children with known routes of infection, 112 (36%) presented with lymphadenopathy. When grouped by the likely route of infection, there was a significantly higher incidence of lymphadenopathy in orally infected children as compared to those likely infected by contact with animals (43% vs. 29%, Table 1).

**Table 1:** Incidence of lymphadenopathy in 307 children (up to 14 years) diagnosed for brucellosis and with known routes of infection.

	Lymphadenopathy	No lymphadenopathy	total
Disease acquisition via ingestion	71 (43%)	96 (57%)	**167**
Disease acquisition via contact with animals	41 (29%)	99 (71%)	**140**
**total**	**112 (36%)**	**195 (64%)**	**307**

Analysis for significance of differences using Chi-square test yielded Χ^2^ = 5.752 and p = 0.016.

From all children with known infection routes suffering from lymphadenopathy, 108 (96%) presented with lymphadenopathy of the cervical lymph nodes (CLN). In 85 children (76%), CLN were the only lymph nodes affected whereas in the remaining cases, they were affected in the context of a general lymphadenopathy. Interestingly, there was a significantly higher percentage of patients with cervical lymph nodes being the only ones affected among children that had acquired brucellosis by ingestion compared to those infected by contact with animals (83% vs. 63%, Table 2).

**Table 2:** Incidence of cervical lymphadenopathy with cervical lymph nodes being the only ones affected (CL) and general lymphadenopathy with lymph nodes other than CLN affected (GL) in children infected with *Brucella* by ingestion or by contact with animals.

	CL	GL	total
Disease acquisition viaingestion	59 (83%)	12 (17%)	**71**
Disease acquisition viacontact with animals	26 (63%)	15 (37%)	**41**
**total**	**85 (76%)**	**27 (24%)**	**112**

Analysis for significance of differences using Chi-square test yielded Χ^2^ = 5.504 and p = 0.019.

These data suggest that CLN may be involved during human infection with *Brucella* by the oral route.

### *Brucella* specifically targets cervical lymph nodes during oral infection of mice

The most commonly route of *Brucella* infection used in research is the intraperitoneal injection [12]. By administering bacteria directly into the peritoneal cavity, however, initial events of bacterial invasion and onset of infection are bypassed. In order to study the role of CLN more closely in an appropriate mouse model of oral infection, we established a route of infection of mice where *Brucella* is administered directly into the oral cavity.

To compare bacterial colonization of organs following this route of infection to inoculation routes that have been well studied, wild type mice were inoculated with *B*. *melitensis* by the intraperitoneal (with 10^6^ bacteria per mouse), by gavage or by the oral mode (both with 10^9^ bacteria per mouse). After 8 days, mice were sacrificed and the bacterial load of several organs was determined by plating homogenized tissues onto nutrient agar.

**Fig 1:**
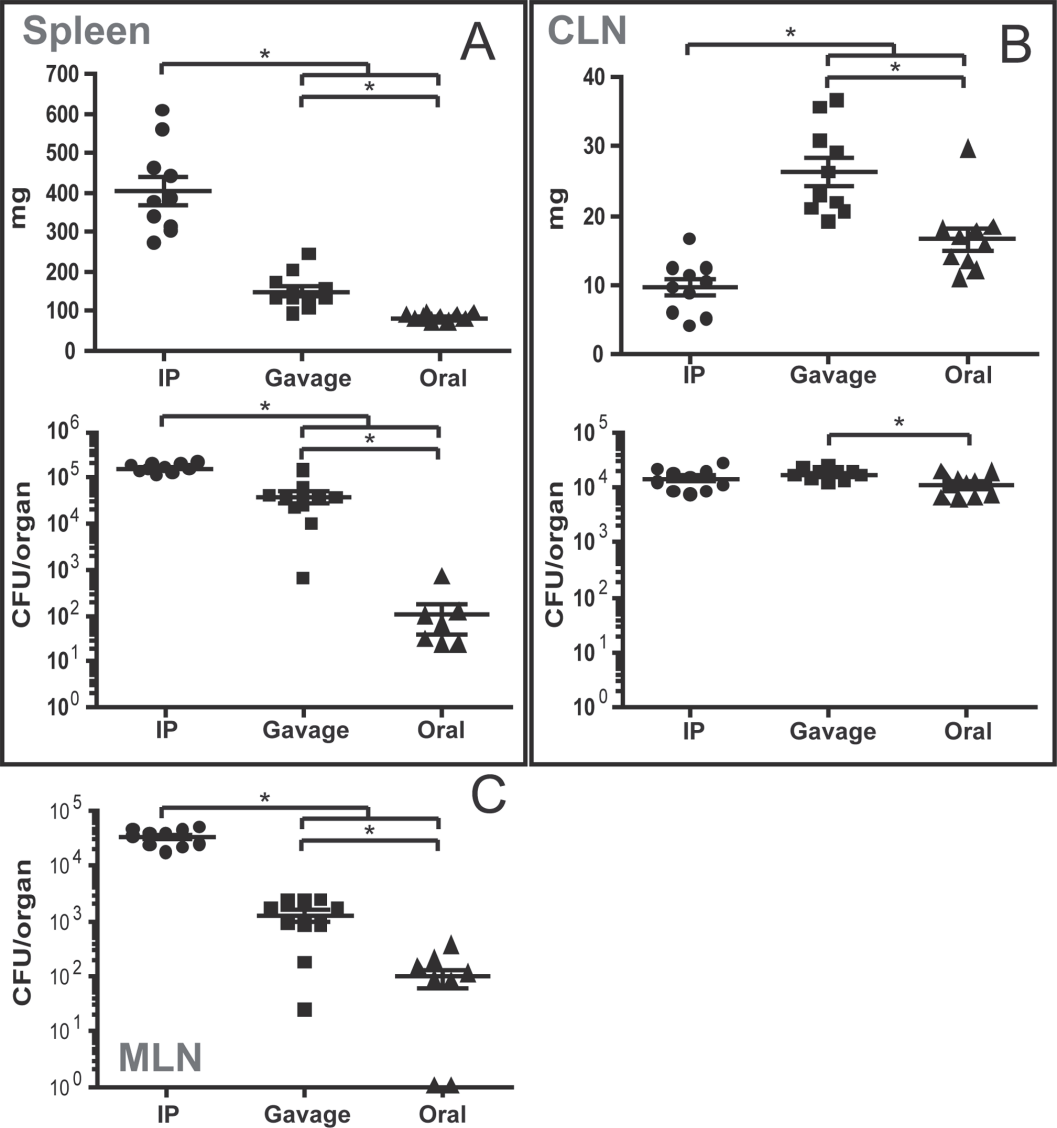
Infection by the oral route leads to preferential bacterial colonization of the CLN. C57BL/6 mice were infected by intraperitoneal injection (10^6^ bacteria/mouse), intragastric by gavage or by the oral route (both at 10^9^ bacteria/mouse). At 8 days post-infection, mice were sacrificed and organs weighed and analyzed for their bacterial loads by plating homogenates on nutrient agar. Data represent mean organ weight or colony forming units (CFU) per organ and SEM of the pooled results from two independent experiments with 5 mice per group. Non-infected organs are not shown due to logarithmic scale. * p ≤ 0.05. CLN—cervical lymph nodes; MLN—mesenteric lymph nodes.

As previously described [12], inoculation into the intraperitoneum (i.p.) resulted in the highest bacterial loads in organs closest to the site of infection, in spleen and mesenteric lymph nodes (MLN) (Fig 1A and 1C). It caused a dramatic increase in spleen weight to an average of 405 +/- 35.0 mg. Inoculation by gavage resulted in spleen and MLN bacterial loads and an average spleen weight that were lower than those of i.p. infection (Fig 1A and 1C). Spleens and MLN after oral infection were colonized at low levels or not at all and no splenomegaly could be observed at the time of sacrifice (Fig 1A and 1C). Bacterial numbers in the CLN, however, were comparable following all routes of infection with moderate significant or no differences depending on the considering the total bacterial load (Fig 1B) or integrating the corresponding organ weight, respectively (S1B Fig). In contrast to i.p. injection and gavage, the oral route of infection resulted in a rather specific bacterial colonization of the CLN. A clear increase of CLN weight could be observed in gavage (26.3 mg +/- 2.0), somewhat less in oral infection (16.6 mg +/- 1.6), whereas CLN weight was lowest following i.p. inoculation (9.6 mg +/- 1.2). Bacterial numbers during i.p. injection were significantly higher in the MLN as compared to the CLN. This relation was inversed for both, gavage and oral infection, where considerably more brucellae were found in the CLN as compared to MLN.

**Fig 2:**
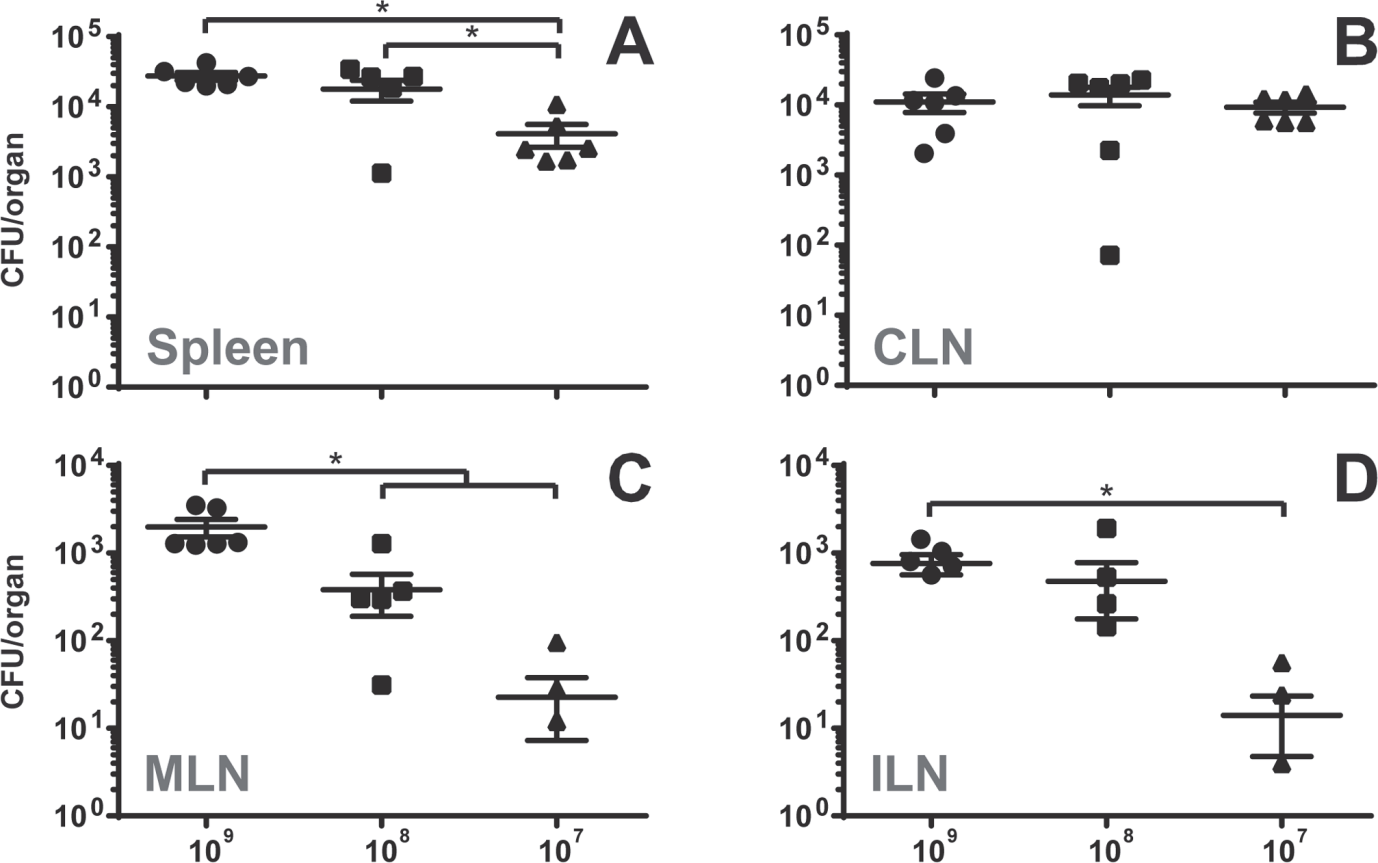
Infection with decreasing bacterial numbers by gavage results in increasingly specific bacterial colonization of the CLN. C57BL/6 mice were infected by gavage with 10^9^, 10^8^ or 10^7^ *B*. *melitensis* per mouse. At 8 days post-infection, mice were sacrificed and organs weighed and analyzed for their bacterial loads by plating homogenates on nutrient agar. Data represent mean CFU per organ and SEM of the pooled results from two independent experiments with 3 mice per group. Non-infected organs are not shown due to logarithmic scale. * p ≤ 0.05. CLN—cervical lymph nodes; MLN—mesenteric lymph nodes; ILN—inguinal lymph nodes.

Doubts have been raised concerning the accuracy of gavage infection [12]. The technique of using rather large volumes that are introduced using a feeding needle are thought to result not just in intragastric infection, but also in infection through tissues of oesophagus and oral cavity through reflux of the inoculum. The preferential colonization of CLN compared to the MLN during gavage infection suggested that bacterial entry does indeed not mainly occur in the intestinal tract but regions of the digestive tract anterior to the stomach. When the inoculum used for gavage infection was titrated down from 10^9^ to 10^8^ or 10^7^ bacteria per mouse, colonization significantly decreased in all organs analyzed except in the CLN (Fig 2), indicating that CLN are more specifically targeted by *Brucella* at low bacterial inocula.

### CLN represent the first location of infection and multiplication during oral infection

**Fig 3:**
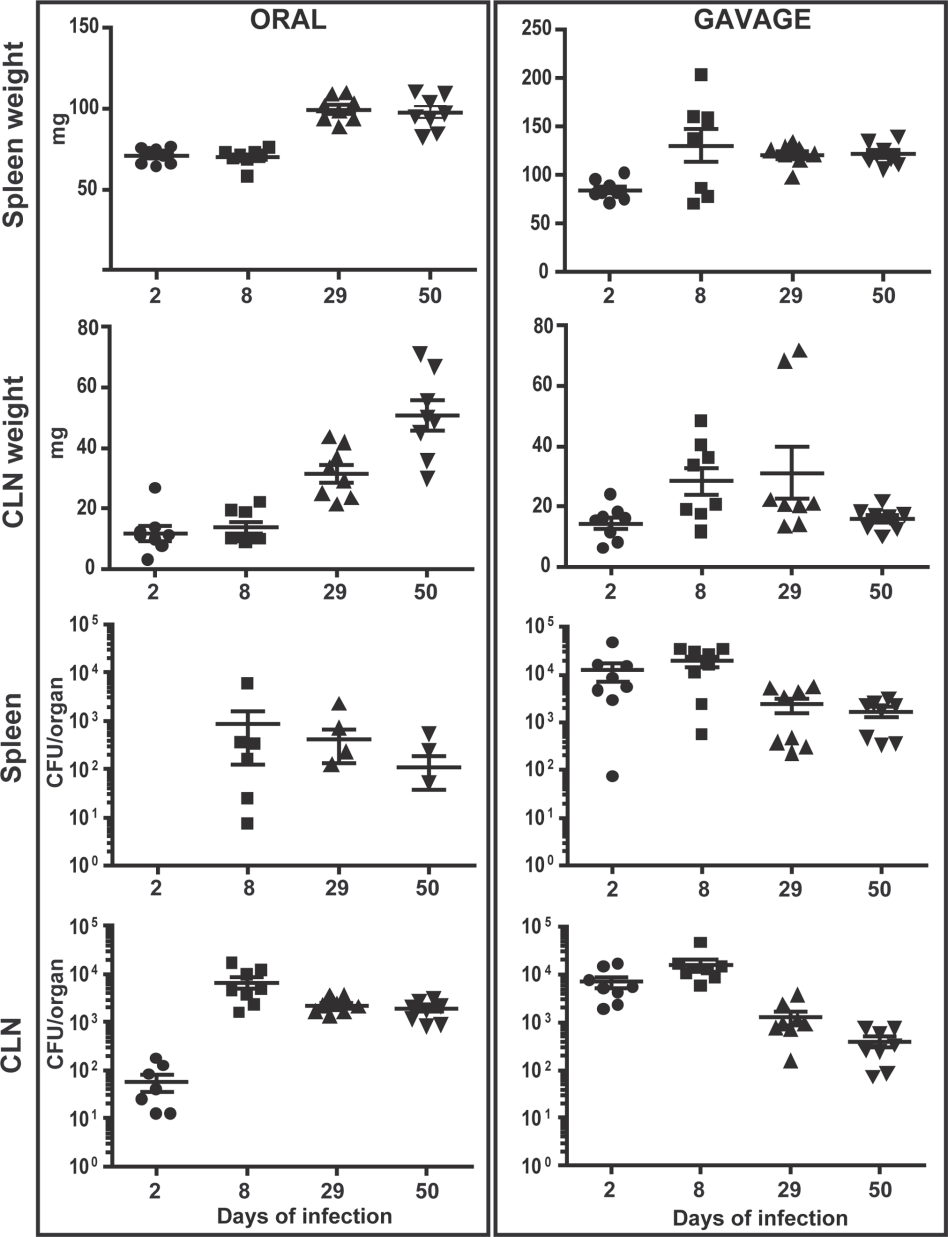
*Brucella* specifically targets and multiplies in cervical lymph nodes during oral infection and causes long-term lymphadenopathy. C57BL/6 mice were infected by gavage or by the oral route with 10^9^ *B*. *melitensis* per mouse. At 2, 8, 29 or 50 days post-infection, mice were sacrificed and organs weighed and analyzed for their bacterial loads by plating homogenates on nutrient agar. Data represent mean organ weight or CFU per organ and SEM of the pooled results from two independent experiments with 4 mice per group.

To study the progress of oral vs. gavage infection by *B*. *melitensis* over time, organs of infected mice were analyzed at different time points post-infection up to 50 days. Following gavage, the organs containing the highest bacterial loads earliest were CLN and spleen (Fig 3). Bacterial numbers in these organs decreased thereafter and stabilized on a plateau up to the last time point analyzed. All other tissues investigated including MLN, ILN and thymus where infected later and at lower levels (S2 Fig). Bacterial multiplication in these tissues showed similar kinetics of an approximate one log increase of bacterial numbers from day 2 to day 8, followed by a constant decrease (S2 Fig). Infection by the oral route also led to an early colonization of the CLN, albeit at lower levels as compared to gavage. However, bacterial numbers in these lymph nodes increased more than 2 logs from day 2 to day 8, leveling up with those in the CLN of gavage infected mice (Fig 3). Thereafter, bacterial counts decreased three fold and remained at this level up to day 50 of infection. In contrast to gavage infection, however, the CLN were the only organs consistently and highly infected during oral inoculation. Infection of other tissues occurred, but randomly (Figs 3 and S2). Whereas the weight of CLN during gavage infection increased in the first 8 days, followed by their slow decrease in mass, the weight of CLN in oral infection continuously increased up to day 50 up to a weight of 50.7 mg ± 5.0 (Fig 3). The mass of spleens of intragastric infected mice increased by a factor of 1.5 from day 2 to day 8 to 130.5 ± 16.8 and then stabilized from day 29 until the end of infection (121.9 ± 4.0 mg at day 50). In contrast, the spleen weight of orally infected mice first significantly increased at 29 days to 99.6 ± 7.4 mg and did not significantly change thereafter up to day 50 (97.5 ± 10.4 mg).

**Fig 4:**
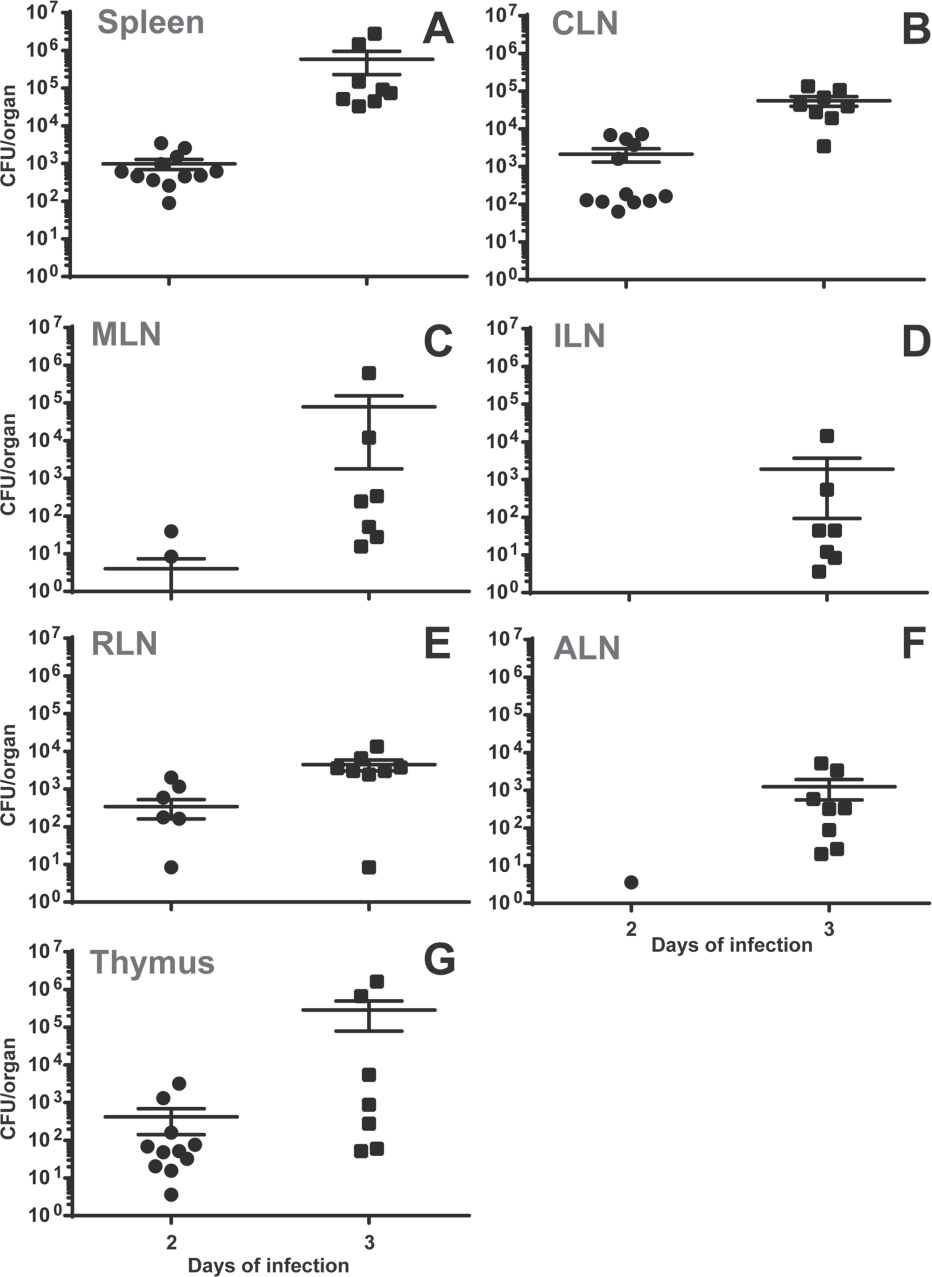
During gavage infection with *S*. Typhimurium, bacteria colonize CLN, spleen and thymus at early time points. C57BL/6 mice were infected with 10^5^ *S*. Typhimurium by gavage. After 2 or 3 days post-infection, mice were sacrificed and organs analyzed for their bacterial loads by plating homogenates on nutrient agar. Data represent mean CFU/organ and SEM of the pooled results from three (day 2) or two (day 3) independent experiments with 4 mice per group. ILN—inguinal lymph nodes; RLN—retropharyngeal lymph nodes; ALN—axillary lymph nodes.

**Fig 5:**
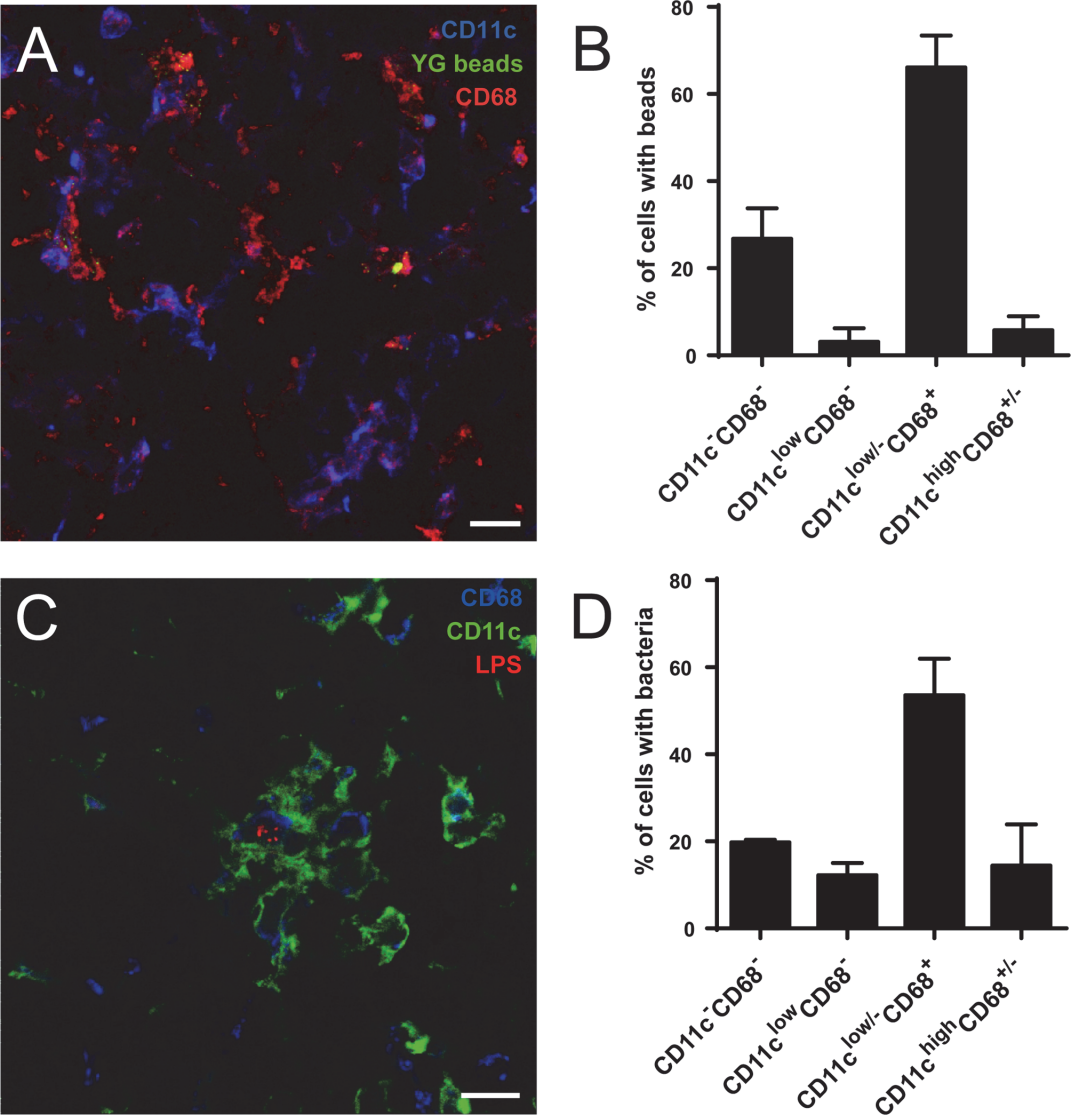
*Brucella* and fluorescent microspheres in the CLN localize in cells positive for CD68 and low or negative for CD11c. **(A)** C57BL/6 mice were fed by oral gavage with 0.2 μm yellow green fluorescent microspheres. After 3 days, they were sacrificed and CLN processed for immunofluorescence microscopy. **(B)** Cells with internal beads from experiments as shown in (A) were quantified as to their expression of CD11c and CD68. At least 100 bead-containing cells per experiment were counted. **(C)** Mice infected by the oral route with 10^9^ *B*. *melitensis* per mouse were sacrificed at day 8, CLN prepared for immunofluorescence analysis as described above and **(D)** the number of infected cells positive for either marker was determined. All available cuts from the CLN of one mouse were analyzed. Data shown represents mean and standard deviation of three independent experiments. Bars: 10 μm.

**Fig 6:**
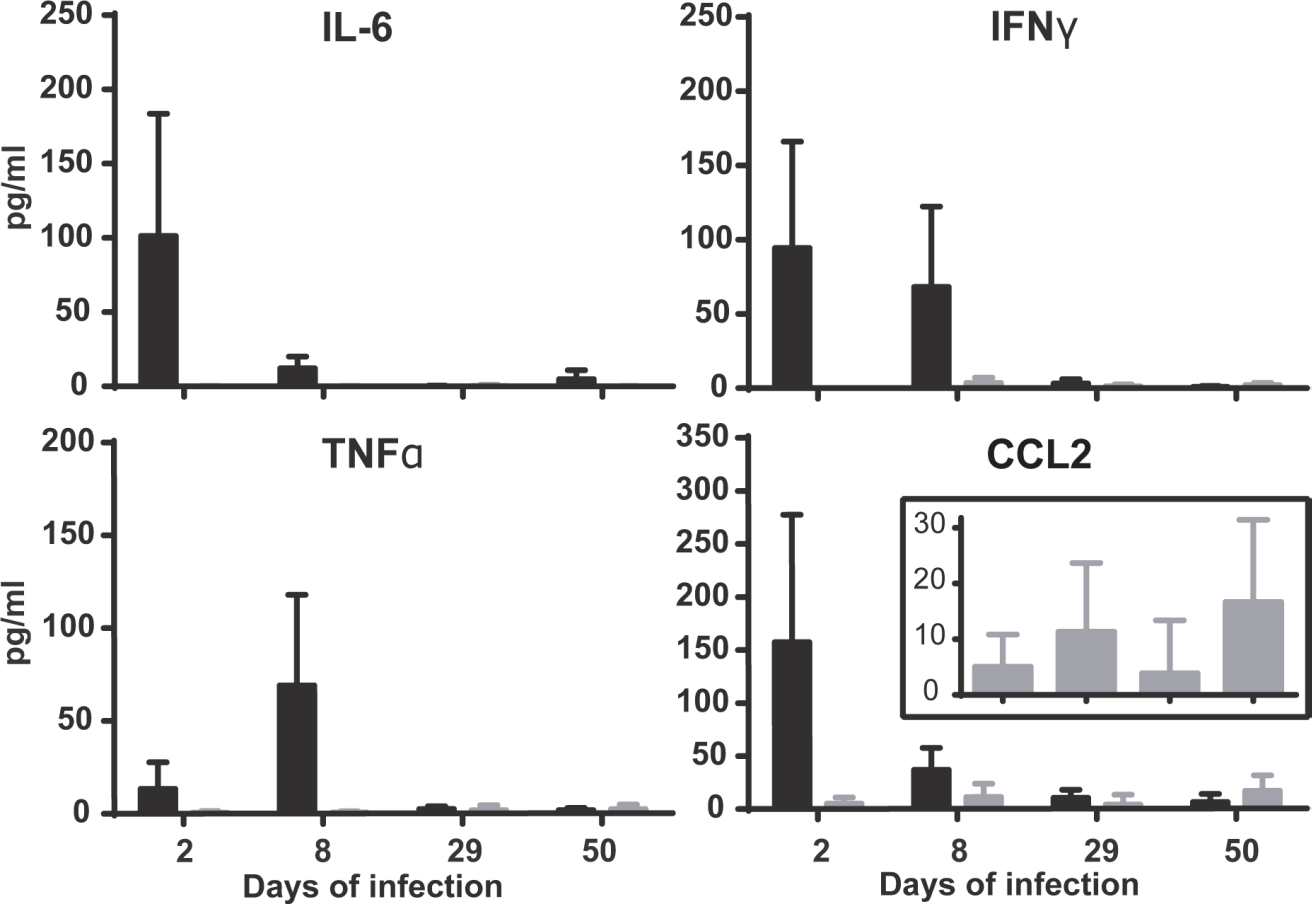
*Brucella* oral infection does not result in secretion of blood cytokines. C57BL/6 mice were infected with 10^9^ *B*. *melitensis* per mouse by gavage or by the oral route. At 2, 8, 29 or 50 days post-infection, blood of mice was recovered and analyzed for the presence of cytokines. Data represent mean and standard deviation from results of two independent experiments with 4 mice per group. Black bars—gavage infection; grey bars—oral infection.

In order to check for a possible role of migratory antigen-presenting cells in shuttling of bacteria from their point of entry to the CLN, we analyzed infection progress in different knock out models. Chemokine receptors CCR2 or CCR7 are crucial for efficient egress of monocytes from the bone marrow and their recruitment to inflammatory sites [13] or particularly for migration of T cells and dendritic cells into lymph node tissues [14], respectively. However, neither in orally infected mice with deficiencies in chemokine receptors CCR2 or CCR7 nor with temporary depletion of CD11c^+^ dendritic cells or CD207 (langerin)^+^ Langerhans cells did we observe a striking impact on the presence of bacteria in CLN (S3 Fig and Figs 4, 5 and 6).

### Bacterial species other than *Brucella* and microspheres given by gavage are efficiently drained to the CLN

*Brucella* early colonization of CLN could be specific for these bacteria or might represent a general pathway of uptake. To check for this, mice were infected with 1x10^5^ of the enteropathogen *Salmonella enterica* Serotype Typhimurium 12023 by either the oral route or by gavage. Organs were harvested after 2 or 3 days of infection and analyzed for their bacterial burden. Interestingly, nearly no bacteria could be recovered following the oral challenge with *Salmonella*. Following gavage infection, however, the first organs to be infected most consistently and at highest levels were CLN, spleen and thymus (Fig 4). Comparing CFU per gram tissue to take into account the difference in weight of the respective organs, bacterial load in the CLN was 1 log higher than that in the spleen at day 2 post-infection (S7 Fig). At day 3, *Salmonella* had disseminated to every organ analyzed and bacterial burden in CLN and spleen had come to equal levels (Fig 4).

A similar prominent and early drainage to the CLN such as that of both bacterial species tested could be observed for 0.2 μm fluorescent microspheres that were found at high numbers 3 days after gavage (Fig 5A). Drainage to the CLN therefore appears to be a more general pathway of material delivered by the oral route.

### Within CLN, microspheres and *Brucella* reside predominately in CD68^+^CD11c^low/-^ cells

To analyze the precise localization of particles drained to the CLN, thin sections of these lymph nodes from mice inoculated orally or by gavage were analyzed by immunofluorescence microscopy. Sections were stained with antibodies against CD11c and CD68 (macrosialin), a glycoprotein that has been shown to be highly expressed in macrophages and at lower levels in dendritic cells [15]. Tissues of *Brucella*-infected mice were additionally stained with antibodies against *Brucella* LPS to detect bacteria.

The large amount of polystyrene beads found in the CLN at 3 days after gavage mostly (66.1 +/- 7.3%) concentrated to frequently high numbers in cells positive for CD68 and low or negative for CD11c (Fig 5A and 5B). Since a certain number of bacteria is required to be able to visualize infected cells in histology, *Brucella* in infected CLN were analyzed at the peak of their bacterial load at eight days after oral inoculation. At that time, 53.6 +/- 8.4% of *Brucella*-infected cells were CD68^+^CD11c^low/-^ (Fig 5D). These cells mostly showed a high level of autofluorescent intracellular vesicles in epifluorescence microscopy and likely represented macrophages. 14.4 +/- 9.5% of *Brucella*-infected cells were CD11c^high^CD68^low/-^, 12.2 +/- 2.8% CD11c^low^CD68^-^ and 19.7 +/- 0.6 of infected cell were negative for both markers (Fig 5D). The largest part of *Brucella*-infected cells was found in the paracortex and they were frequently associated in clusters with CD11c^high^ cells (Fig 5C).

### Oral *Brucella* infection does not induce an increase in serum cytokines

Having identified CLN as the main location of *Brucella* replication during oral infection, we sought to analyze the systemic response. To this aim, mice were inoculated by the oral route or by gavage and their sera analyzed for the presence of cytokines at different time points. Gavage infection resulted in high levels of IFNγ, CCL2 and IL-6 already at day 2, followed by the gradual decrease of these cytokines (Fig 6). TNFα was first found to be substantially increased at day 8 and decreased to the limit of detection at 29 days post-infection (Fig 6). In contrast, oral infection did not trigger secretion of any of these cytokines at any time point with exception of CCL2 that could be detected at low levels (Fig 6).

### Oral infection with *Brucella* induces a local inflammatory response in the CLN

**Fig 7:**
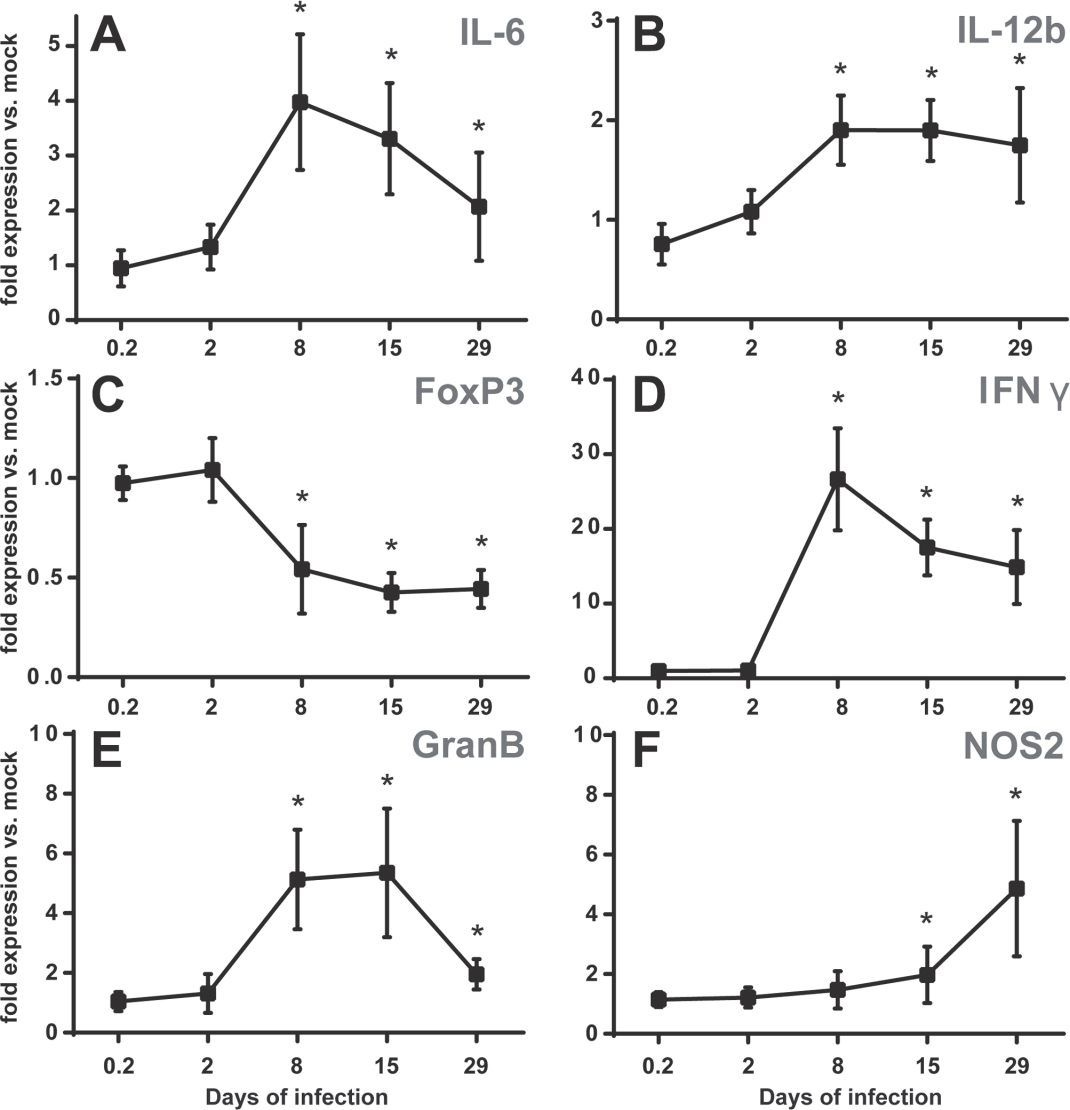
Oral infection with *B*. *melitensis* induces pro-inflammatory gene expression in the CLN. C57BL/6 mice were infected with 10^9^ *B*. *melitensis* per mouse or mock infected by the oral route. At 5 h, 2, 8, 15 or 29 days post-infection, mice were sacrificed, total RNA of the CLN was extracted and analyzed for expression of genes involved in inflammatory responses by reverse transcription real-time PCR. Results are given as fold expression compared to the signal obtained for mock-infected mice. Data represent means and standard deviations of two independent experiments with 3 or 4 mice per group. * p ≤ 0.05 as compared to mock infected expression levels.

In spite of the absence of pro-inflammatory cytokines in the blood, the increase of lymph node weight during oral infection suggested a strong local inflammatory reaction. To analyze the type of response, CLN of mice orally infected with *B*. *melitensis* were harvested at different times post-infection. Their total RNA was isolated and the expression of several genes known to be involved in inflammation was determined by quantitative real time PCR and analyzed as fold expression of that in mock-infected controls. A clear response in gene expression could be observed starting from day 2 and reaching a peak at day 8 post-infection (Fig 7). The high expression levels of IL-6 and IFNγ declined after day 8 (Fig 7A and 7D), whereas expression of IL-12b and granzyme B remained high up to day 15 before decreasing (Fig 7B and 7E). The up-regulation of pro-inflammatory gene expression was paralleled by a decrease in transcription levels of FoxP3 that stayed between 0.4 and 0.5 from day 8 to day 29 post-infection (Fig 7C). Expression of nitric oxide synthase NOS2 increased with slower kinetics but continuously (Fig 7F). Since IL-12 and NOS2 are strongly, though not exclusively, expressed in activated macrophages, expression levels of several additional genes determining or resulting from particular macrophage polarization were analyzed at day 15 post-infection. Total CLN RNA indicated an up-regulated expression of prostaglandin-endoperoxide synthase 2 (Ptgs2), indoleamine 2,3-dioxygenase 1 (IDO1) and a down-regulation of IL-4 and Krüppel-like factor 4 (Klf4), rather indicating an environment of classically activated phagocytes infiltrating in response to an increased expression of CC-chemokine ligand 2 (CCL2) (S8 Fig). There were only slight changes in expression of IL-10 and osteopontin throughout the entire time course (S9A Fig). Expression levels of TNFα did not change at all, whereas expression of IL-18 was slightly lower as compared to the mock-infected control beginning from day 8 and stayed low until the last time point analyzed (S9D Fig). Overall, the gene expression of CLN in orally infected mice was consistent with a robust inflammatory response.

**Fig 8:**
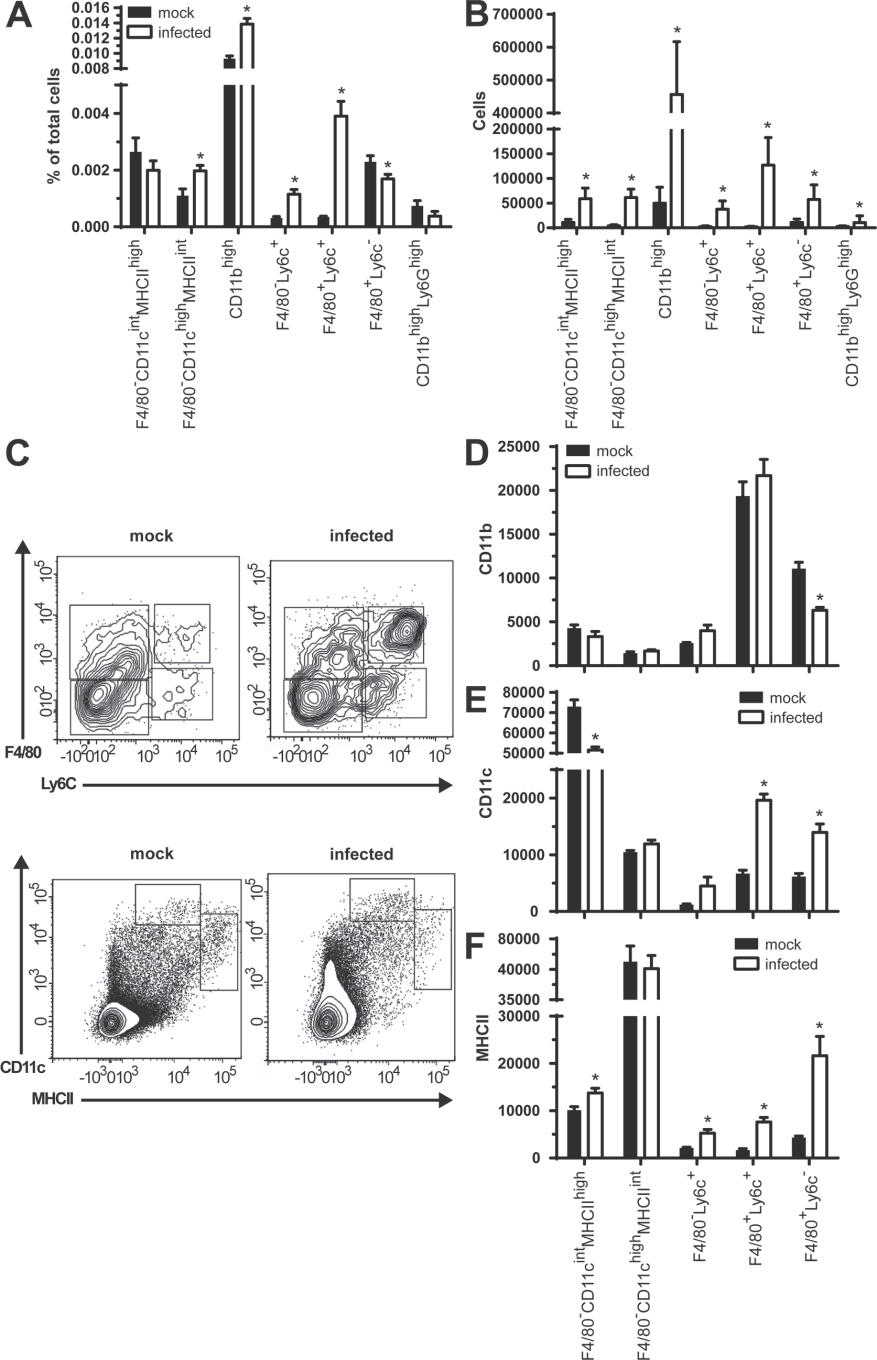
*B*. *melitensis* oral infection results in an increase of CLN CD11b^high^ cells expressing either F4/80, Ly6c or both. CLN from mice orally infected with 10^9^ *B*. *melitensis* per mouse for 15 days were prepared for flow cytometry. Total CLN cell numbers were analyzed for the respective percentages **(A)** or absolute numbers **(B)** of dendritic cells (F4/80^-^CD11c^high^MHCII^int^ and F4/80^-^CD11c^int^MHCII^high^), CD11b^high^ macrophages/monocytes (F4/80^-^Ly6c^+^, F4/80^+^Ly6c^+^ and F4/80^+^Ly6c^-^) and neutrophils (CD11b^high^Ly6G^+^). **(C)** shows a representative contour plot of respective populations from a mock-infected or *Brucella*-infected mouse on CD19^-^Ly6G^-^CD11b^high^ (F4/80 vs. Ly6c) or CD19^-^CD11b^low/int^F4/80^-^NK1.1^-^ cells (CD11c vs. MHCII). Populations shown in (A) were analyzed for their median fluorescence of **(D)** CD11b, **(E)** CD11c and **(F)** MHCII. Data represent mean and SEM of pooled results from two independent experiments with a total of 8 (mock-infected) and 9 (infected) mice per group. * p ≤ 0.05 as compared to respective mock infected control.

To complement analysis of gene expression, we investigated cell populations present in CLN by flow cytometry. Mock or *B*. *melitensis* orally infected mice were sacrificed at 15 days post-infection when the pronounced CLN hyperplasia first became evident. CLN were harvested and were prepared for flow cytometry analysis. Most striking changes observed concerned macrophages and dendritic cells. *Brucella* infection led to an increase in percentages of F4/80^-^CD11c^int^MHCII^high^, F4/80^-^Ly6c^+^ and most prominently of F4/80^+^Ly6c^+^ cells, whereas relative numbers of F4/80^+^Ly6c^-^ cells decreased (Fig 8A and 8C). In all CD11b^high^ cells positive for either Ly6c or F4/80, or both, there was a clear increase in expression of CD11c and MHCII (Fig 8E and 8F), whereas only in F4/80^+^Ly6c^-^ cells a decrease in surface CD11b could be observed. Although the absolute numbers of all cell types analyzed considerably increased upon infection (Fig 8B and S10E), we did not observe any change in relative numbers of CD8^+^ or CD4^+^ T lymphocytes, even though there were minor shifts in their CD44 and CD62L expressing subpopulations (S10A and S10B Fig). The percentage of CD19^+^ B lymphocytes however was characterized by a clear increase (S10A Fig). In B as well as T lymphocytes we observed a decrease in mean CD62L and CD44 fluorescence (S10C and S10D Fig). Overall these data show that CLN are subject to massive recruitment and/or proliferation of inflammatory cell populations such as macrophages/monocytes, B and T lymphocytes in orally infected mice.

### CLN of orally inoculated mice develop small and multifocal granulomas

**Fig 9:**
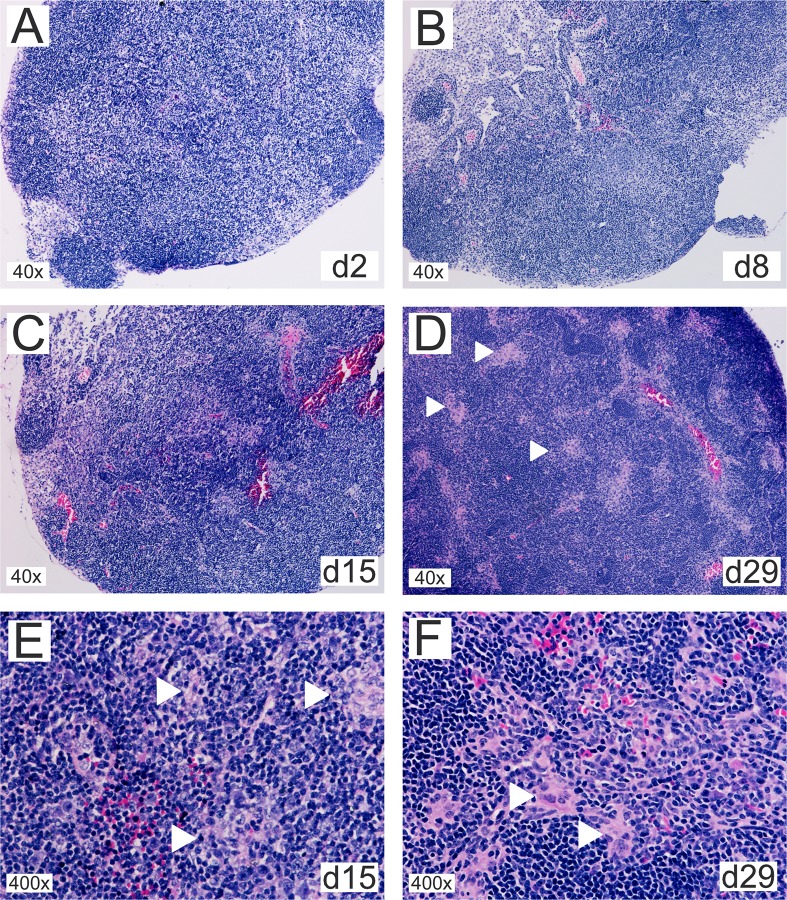
Oral infection with *B*. *melitensis* results in CLN granuloma formation. Thin sections of cervical lymph nodes from mice orally infected with 10^9^ *B*. *melitensis* per mouse for (A) 2, (B) 8, (C) 15, or (D) 29 days were stained with eosin-hematoxylin. (E) and (F) show higher magnifications from day 15 and 29, respectively. White arrowheads mark granulomatous structures that (E) develop as multifocal loose cell arrangements that (F) gradually solidify into compact granulomas composed of epithelioid cells with occasional multinucleated giant cells and few neutrophils.

To integrate results obtained by transcriptional and flow cytometry analysis in the context of whole tissues, the inflammatory response in the CLN of orally infected mice was investigated using classical eosin/hematoxylin staining. From early stages on (S11 Fig) up to day 2 post-infection, no changes in lymph node structure could be observed apart from occasional mild germinal center development (Fig 9A). At 8 days post-infection, an infiltration of histiocytes could be observed in the paracortical area with no to mild expansion of the paracortex and occasional secondary follicles (Fig 9B). The paracortical histiocytic infiltrations developed to multifocal loose granulomatous arrangements at 15 days post-infection (Fig 9C and 9E). At day 29, these structures became increasingly compact to form multiple small, preferentially perivascular granulomas in the paracortex that at this stage was characterized by pronounced hyperplasia (Fig 9D and 9F). Granulomas were mainly composed of epitheloid cells, few multinucleated giant cells and rarely neutrophils. At day 50, there was some but moderate evidence of necrotic debris inside of the granulomatous foci (S11 Fig). The hyperplasia of the paracortex persisted up to this time of infection.

Results suggested that even though oral infection remains mostly silent in terms of systemic reactions, CLN show a robust local response to *Brucella* invasion.

## Discussion

Many pathogens, particularly viruses but also protozoa and bacteria, have been described to enter through the oral mucosa and to be drained to CLN [1]. *Toxoplasma* infection might as well be the reason for cervical lymphadenopathy [16] as extrapulmonary tuberculosis which mostly affects lymph nodes and of these most frequently the CLN [17,18]. Especially in children, cervical lymphadenitis is a common phenomenon [19], likely reflecting the constant exposure of these organs to foreign material drained from mucosa tissue of the head in a developmental stage when the immune system is still in development.

Little is known as to the role of CLN in human brucellosis. However, consumption of non-sterilized milk products is generally an important source of infection, especially for urban populations [9]. In 1956, Spink described lymphadenopathy as “the third most common sign” [20] in human brucellosis that occurs between ¼ to slightly more than ^1^/_3_ of cases. Lymph nodes most commonly affected are CLN and axillary lymph nodes (ALN) and the handling of infected material with ungloved hands has been correlated to ALN involvement [20]. However, in recent studies involvement of lymph nodes is a matter of discussion. One reason for discrepancies may be the fact that patients are analyzed in groups according to the focus of the article like sex [21], age [22,23], illness duration (acute vs. chronic) [24], form (with vs. without complication) [25] or *Brucella* species [26,27], but rarely with respect to the likely source of infection. Moreover, these sources may not always be easily determined, particularly in regions where brucellosis is endemic. The actual location of lymphadenopathy is often disregarded, if lymph nodes are analyzed at all. However, bacteria could actually be found in lymph nodes of patients by cultivating lymph node aspirates [20], but to our knowledge and possibly because this method does not make part of standard diagnostic procedures, it has not been analyzed large-scale.

Increased incidence of (cervical) lymphadenitis in a rather homogenous group of patients comprising children less than 14 years old infected with *Brucella* by the ingestion led us to develop a murine model for oral *Brucella* infection. Using this infection mode, most bacteria were found in the CLN and only occasionally disseminated to other organs. In experimental conjunctival and naturally occurring infection of cattle, *Brucella* have been found in lymph nodes of the head to a similar extent [28,29]. Upon arrival in the lymph nodes, bacteria are thought to be captured or to disseminate further depending on the resistance properties of the individual and the strain’s virulence [30]. These results support the notion of lymph nodes serving not only as a site for antigen presentation to mount responses but also as an initial guarding gate for invaders. Phagocytes in regional lymph nodes have been demonstrated to trap arriving particles preventing their systemic spread and subsequently orchestrate efficient responses involving an “innate CD8” population providing antigen-independent IFNγ secretion [31].

However, CLN may not only serve as the first tissues to filter and trap bacteria and to prevent further dissemination, but maybe also as long-term reservoir. Brucellae are known to persist immunologically silent in some tissues over long periods of time [28]. Lymph node persistence has also been shown for *Salmonella*. In a model for chronic infection in Nramp1-positive mice, *S*. *typhimurium* is contained for up to 1 year in the MLN and infection can be reactivated by depletion of IFNγ [32], a cytokine also crucial for control of *Brucella* infection [33].

Generally being regarded as an enteropathogen, we found that also *Salmonella* is efficiently drained to the CLN upon gavage infection where bacteria appear earlier and in higher numbers than in mesenteric lymph nodes and spleen. Even though infection later on spreads to any organ, it supports the view of gavage representing a mixed infection mode of intragastric and oral and that *Salmonella* are able to follow the same route as *Brucella* even though somewhat less efficient, since infection by oral mode using smaller volumes did not result in host colonization. *Salmonella* has been observed in the CLN in several studies and it has been found that they reach these lymph nodes travelling free in the lymph or associated with granulocytes and macrophages [34,35].

We did not observe any systemic response following *Brucella* oral infection, but bacteria initiated a local inflammation in the CLN themselves, particularly a marked up-regulation of IFNγ and an infiltration of macrophages/monocytes whose capacity to eliminate intracellular *Brucella* was shown to considerably increase upon IFNγ exposure [36–38]. However, even though this response seems sufficient to limit dissemination, it does not readily eradicate bacteria. Besides the particular adaptations of *Brucella* as intracellular pathogen, one reason for this impasse might be the formation of granulomas that prevent spreading of *Brucella* but also provide a protected environment for pathogens to withstand the microbicidal arsenal of immune cells [39]. Granulomas in brucellosis have been shown to occur in various tissues and are of the epithelioid type [40], with CD11b^+^, F4/80^+^, MHCII^+^ cells expressing high levels of Nos2 [41] in agreement with our findings. The dual role of macrophages being crucial to limit and contain bacterial in the onset of infection and serving as host cell in later stages may also explain the higher bacterial load obtained in CLN of CCR2 knock out mice that have decreased numbers of peripheral monocytes.

*Brucella*-infected macrophages *in vitro* have been demonstrated to mount a pro-inflammatory response including induction of IL-6, Ptgs2, Nos2 and TNFα [42,43]. Even though TNFα depletion using antibodies exacerbates bacterial burden in mice [44], human macrophages lack induction in TNFα secretion after *Brucella* infection [45] and intraperitoneal injection of mice with the bacteria leads to a minimal TNFα serum concentration [46]. In line with this, we observed no visible change in TNFα expression in *Brucella*-infected CLN. There was a slight but significant reduction of IL-18 expression starting from day 8. IL-18 is known to enhance IFNγ expression in synergy with IL-12 and to directly induce secretion of pro-inflammatory cytokines [47]. Re-challenged macrophages and splenocytes from *Brucella*-infected mice produce less IL-18 than those of uninfected mice without noticeable reduction of secretion of IFNγ and might be the result of active limitation of IL-18 secretion by *Brucella* [48]. Expression of pro-inflammatory genes as well as down-regulation of FoxP3 reached their maximum levels at day 8, followed by either their stabilization or their gradual returning to normal levels. The delayed increase of Nos2 expression coincided with a decline in IFNγ at the onset of the chronic phase. Nos2 is expressed in multiple cells, but its up-regulation also makes essential part of the typical expression signature of (murine) activated macrophages [49].

In contrast to oral infection, inoculation by gavage led to colonization of several organs as it has been shown before [50], yet organs infected at highest level earliest were CLN and spleen. Results obtained for gavage inoculum titration suggest that 1) gavage results in a mixed infection mode involving the oral and intragastric routes, 2) that bacterial entry into tissues drained by CLN represents the most successful passage into the host, supporting the notion of *Brucella* is not an enteropathogenic bacterium [51] and that 3) even during gavage infection, the initially draining tissue are the CLN, filtering particles before reaching any other organ and letting only pass what exceeds capacities of local macrophages. The higher overall infection level using gavage could result from a higher volume of inoculum leading to more thorough and prolonged contact of mucosal tissues with infectious liquid as in oral infection. Effects may be even more pronounced due to the anesthesia of mice, resulting in reflux, absence of swallowing reflex and exacerbation of disease by ketamine [52]. Intriguingly, many of the *Brucella* natural hosts, including cows, sheep, goats and camels, are ruminants. In these animals, ingested food will circulate between non-acidic stomach compartments before regurgitation, leading to intense contact of potentially infected material with the oral mucosa before being passed on to the acidified abomasum. Drainage to CLN therefore may represent a particular adaptation and general first site of infection by *Brucella*, also occurring in non-ruminant species.

Interestingly, infection by gavage with both, *Brucella* and *Salmonella*, also led to bacterial colonization of the thymus. The thymus may be infected by several pathogens, including mycobacteria [53,54] and can also be targeted by *B*. *abortus* after experimental oral infection of wolves [55]. Even though long considered as immune-privileged site, these infections may result in inflammatory responses. Long-term persistence of pathogens in the thymus can lead to a decrease in pathogen-specific T cells and in consequence to a less effective immunity [53,56], which may also be relevant in the context of brucellosis.

The oral mode of infection described here allows the study of bacterial and host factors involved in the initial process of bacterial crossing of epithelial barriers, thereby offering several advantages: It matches more closely to the natural route of entry and initial dissemination routes of bacteria, whereas e.g. injecting bacteria into the intraperitoneum may expose them to several factors and cell types they might never encounter in a situation of natural infection. Oral infection involves a minimum of stress, no wounds and no anesthesia which may impact on experimental results [52]. It may contribute to create a less “splenocentric” view in research of infectious diseases acquired by the oral route. The efficient drainage of ingested material to the CLN suggests an important role for these lymph nodes particularly in onset of infection. As a default interface between outside and inside, draining all mucosal tissues from the head, they likely take a pivotal role mediating either resistance (successful capture and elimination of bacteria) or susceptibility (bacterial systemic spread), the mechanisms of which still require further investigation. That the actual point of entry of bacterial pathogens may determine infection progress is also suggested by the fact that different lymph nodes draining distinct tissues in a healthy organism show different basal level expression profiles of genes involved in inflammation [8,57,58] (S12 Fig).

Together, our results highlight the role of CLN in serving as both, early and efficient trap for orally internalized bacterial invaders and as possible reservoir for chronic pathogens. It has also been speculated that since the lymph nodes of the head and neck are along the natural infectious pathway and the first ones having to react to infection, routes involving those nodes might be an interesting target for vaccines [28]. Reevaluating CLN as an initial checkpoint therefore also hints to a possible significance of oral routes for means of vaccination.

## Material and Methods

### Ethics statement

Animal experimentation was conducted in strict accordance with good animal practice as defined by the French animal welfare bodies (Law 87–848 dated 19 October 1987 modified by Decree 2001–464 and Decree 2001–131 relative to European Convention, EEC Directive 86/609). All animal work was approved by the Direction Départmentale des Services Vétérinaires des Bouches du Rhône (authorization number E 13-055-10 delivered for 5 years on 11/30/2012). INSERM guidelines have been followed regarding animal experimentation (authorization No. 02875). Data on human lymph nodes were acquired entirely by retrospective analysis of 23 years-old medical records of as detailed below. Approval from institutional review board (University hospital for infectious diseases and febrile conditions, Medical faculty of Skopje, Macedonia) was given under the IRB number 0302-480/1. Patient records/information was anonymized and de-identified prior to analysis.

### Analysis of lymphadenopathy in brucellosis patients

The group of patients retrospectively analyzed comprised 317 children not older than 14 years old diagnosed with brucellosis at the University Clinic for Infectious Diseases and Febrile Conditions, Skopje, Republic of Macedonia during the period from January 1989 to December 2011. The diagnosis of brucellosis was based on clinical findings compatible with brucellosis (arthralgia, fever, sweating, malaise, hepatomegaly, splenomegaly, signs of focal disease), supported by detection of specific antibodies at significant titers and/or demonstration of at least a fourfold rise in antibody titer in serum samples obtained 3 to 4 weeks apart. Antibody titers were determined by standard tube agglutination (STA), *Brucella* Coombs test, or the Brucellacapt assay. The corresponding titers considered to be positive were ≥1/160, ≥1/320 and >1/320, respectively. During the study period, the bacteriological isolation was not a routine practice in the Republic of Macedonia.

### Bacteria and microspheres

The bacterial strains used in this study were *Brucella melitensis* 16 M with a chromosomal integration of DsRed (denoted here as *B*. *melitensis*) [59], wild type *Brucella abortus* 2308 [60] and *Salmonella enterica* Serovar Typhimurium 12023 [61]. The *B*. *melitensis* was grown in tryptic soy broth containing 25 μg/ml kanamycin at 37°C and 200 rpm for 16 h. *Salmonella* was grown in LB medium for 16 h at 37°C and 200 rpm. *S*. Typhimurium were subcultured in fresh LB at a dilution of 1:100 and grown for 2 h at 37°C. In preparation for infection, bacteria were pelleted, resuspended in PBS and adjusted for the respective MOI used in PBS (for gavage or intraperitoneal) or in 5% milk (Scharlau) that had been heat-sterilized for 20 min at 100°C and cooled down before use (for oral infections). All experiments with live *Brucella* species were performed under biosafety level 3 conditions. Fluorescent beads used in this study were Yellow Green 0.2 μm carboxylated microspheres from Polysciences.

### Animals

Six weeks old female C57/BL6 mice were obtained from Charles River and Janvier and used for experiments not later than three weeks after arrival. All infections with *Brucella* and *Salmonella* were carried out in a biosafety level 3 facility. Mice were obtained from the following sources: CCR2-/- mice [62], CCR7-/- mice [63], Langerin-DTR mice [64] and CD11c-DTR/GFP [65]. For depletion of CD11c-expressing cells, bone marrow chimeras were created: wild type C57BL/WT mice of 7 weeks were lethally irradiated and reconstituted with 4x10^6^ bone marrow cells prepared from CD11c-DTR/GFP mice. After 8 weeks, mice were used for *Brucella* infections. To this aim, they were treated with 150 ng diphtheria toxin 24 h before and 24 h after oral inoculation. Control mice were injected with PBS only. Langerhans cells from Langerin-DT mice were depleted directly by intraperitoneal injection of diphtheria toxin 4 days before and again one day before infection.

### Mouse infections

For gavage infection, mice were anesthetized using 100 μl of rompun-ketamine. Bacteria in PBS were delivered in a volume of 100 μl per mouse using a 1 ml syringe equipped with a feeding needle. Between each mouse, the needle was rinsed with ethanol and twice with PBS. For oral infections, mice were held in scruff and were fed with 10 μl of the bacteria dispersed in 5% heat-sterilized milk (Scharlau) using a standard micropipette. For intraperitoneal infection, bacteria in PBS were injected into the intraperitoneal cavity in a volume of 100 μl. At the end of experiments, mice were sacrificed by cervical dislocation.

### Analysis of organ bacterial burden

Organs were sterilely removed and placed into defined volumes of PBS. Collected lymph nodes were according to the definitions given by Van den Broeck *et al*.[66]: mandibular, accessory mandibular and superficial parotid lymph nodes (herein referred to in their entity as cervical lymph nodes or CLN), jejunal and colic lymph nodes (together referred to as mesenteric lymph nodes or MLN), proper and accessory axillary lymph nodes (together referred to as axillary lymph nodes or ALN), subiliac lymph nodes (referred to as inguineal lymph nodes or ILN), cranial deep cervical lymph node (referred to as retropharyngeal lymph node or RLN). After weight determination of CLN and spleen, tissue and liquid were transferred into 24-well plates and homogenized using the plunger of a sterile syringe. Bacterial counts were determined by plating serial dilutions of homogenized organs onto tryptic soy agar plates.

### Reverse transcription Real-time PCR

All products for RNA isolation and reverse transcription were from Qiagen. Cervical lymph nodes of uninfected or *Brucella*-infected mice were stabilized in RNAlater immediately after harvesting. Tissues were lysed using a pestle, homogenized with QIAshredders and total RNA was extracted with the RNeasy Mini Kit according to the manufacturer’s instructions. Of each sample, 800 ng of RNA were reverse transcribed using the QuantiTect Reverse Transcription Kit. Equal amounts of cDNA were analyzed in RT PCR with the Power SYBR Green PCR Master Mix (Applied Biosystems) in an Applied Biosystems 7500 Fast Real-Time PCR System with hypoxanthinephophoribosyltransferase (HPRT) as reference house keeping gene. Primers used are given in S1 Table.

### Histological analysis by immunofluorescence and immunohistochemistry

For immunofluorescence, samples were fixed in cold 3.2% formaldehyde in PBS for 2h at 4°C, rinsed twice with cold PBS and incubated in 35% sucrose in PBS over night at 4°C. They were embedded and frozen into O.C.T. freezing compound (Sakura) and stored at -80°C until sectioning. Tissues were cut into 20 μm sections. For immunofluorescence staining, samples were permeabilized in PBS/0.5% saponin for 7 min and blocked in PBS/0.1% saponin containing 2% BSA and 1% FCS for 30 min. They were incubated with primary antibodies in PBS/0.1% saponin overnight at 4°C, rinsed, followed by incubation with the secondary antibodies in the same buffer for 2 h at RT. Sections were thoroughly rinsed in PBS and water, embedded in ProlongGold (Invitrogen) and analyzed using a confocal Leica SP5X microscope.

For classical immunohistochemistry, samples were fixed in buffered formol for at least 48 h before embedding into paraffin and sectioning. Thin sections were colored using standard eosin/hematoxylin staining procedures and analyzed using a Nikon H600L microscope.

### Flow cytometry analysis

For flow cytometry analysis, organs were harvested into RPMI/2% FCS containing 337 u/ml collagenase II (Worthington) and 0.14 mg/ml DNaseI (Sigma). They were cut into small pieces and tissues were digested for 30 min at 37°C with occasional resuspension. Of all samples, small volumes were fixed and counted to determine cell numbers. 100 μl of cells were pelleted, washed once in FACS buffer (PBS / 2 mM EDTA and 2% FCS) and stained for 30 min at 4°C in FACS buffer containing antibodies. Cells were washed and resuspended in PBS containing Live/Dead Fixable staining (Invitrogen) and incubated at RT for 12 min. Samples were washed once more and fixed in 3.2% formaldehyde in PBS for 20 min at RT. They were analyzed using a FACS LSRII system (BD Biosciences).

### Antibodies

Antibodies used for immunofluorescence were rat anti-mouse CD68 (Serotec), hamster anti-mouse CD11c (N418, Biolegend), rabbit polyclonal anti-*Brucella* LPS (gift from Edgardo Moreno, Universidad Nacional, Costa Rica).

Antibodies for flow cytometry were MHCII-Alexa700 (eBioscience), CD11b-eFluor 450 (eBioscience), Ly6C-PerCP-Cy5.5 (BD Bioscience), Ly6G-APC-Cy7 (Biolegend), CD11c-PE-Cy7 (Biolegend), F4/80-PE (Biolegend), CD86-FITC, CD8-Alexa700 (BD Bioscience), CD4-APC-eFluor780 (eBioscience), CD19-BV-605 (Biolegend), CD62L-PE-Cy5 (Biolegend), CD5-PE-Cy7 (Biolegend), and CD44-FITC (BD Bioscience). Live cells were distinguished by staining with LIVE/DEAD Fixable Aqua Dead Cell Stain Kit (Life technologies).

### Serum cytokine determination

For the quantification of serum cytokines, the CBA Mouse Inflammation Kit (BD Bioscience) was used following the instructions of the manufacturer.

### Statistical analysis

All data obtained was analyzed using Prism (GraphPad) or Excel (Microsoft). For human analysis, significance was determined using Chi-square test. Data of all remaining experiments were analyzed in unpaired, two-tailed Student’s t-tests. Differences were considered significant with p≤0.05. For analysis of gene expression by RT-PCR, mean values obtained for mock-infected mice were set as one and values obtained for infected mice expressed as fold expression vs. mock.

## Supporting Information

S1 Table Primers used for analysis of gene expression upon oral infection. (DOC)

S1 Fig Infection by the oral route leads to preferential bacterial colonization of the CLN. C57BL/6 mice were infected by intraperitoneal injection (10^6^ bacteria/mouse), intragastric by gavage or by the oral route (both at 10^9^ bacteria/mouse). At 8 days post-infection, mice were sacrificed and organs weighed and analyzed for their bacterial loads by plating homogenates on nutrient agar. Data represent mean of colony-forming units per gram tissue and SEM of the pooled results from two independent experiments with 5 mice per group. Non-infected organs are not shown due to logarithmic scale. * p ≤ 0.05.
(TIF)

S2 Fig Gavage infection with *Brucella* leads to colonization of all organs analyzed, whereas bacteria are isolated from these organs only sporadically after oral inoculation. C57BL/6 mice were infected with *B*. *melitensis* with 10^9^ bacteria per mouse by gavage or by the oral route. At 2, 8, 29 or 50 days post-infection, mice were sacrificed and organs analyzed for their bacterial loads by plating homogenates on nutrient agar. Data represent mean CFU per organ and SEM of the pooled results from two independent experiments with 4 mice per group.
(TIF)

S3 Fig Chemokine receptor CCR2 is not required for bacterial colonization of CLN. CCR2 deficient mice or wild type mice were infected with *B*. *melitensis* with 10^9^ bacteria per mouse by the oral route. At 8 days post-infection, mice were sacrificed and organs analyzed for their bacterial loads. Data represent mean CFU per organ and SEM of results from two independent experiments with three mice per group.
(TIF)

S4 Fig Chemokine receptor CCR7 is not required for bacterial colonization of CLN. Mice with a CCR7 deficiency or wild type mice were infected with *B*. *melitensis* with 10^9^ bacteria per mouse by the oral route. At 8 days post-infection, mice were sacrificed and organs analyzed for their bacterial loads. Data represent mean CFU per organ and SEM of results from two independent experiments with 3 mice per group.
(TIF)

S5 Fig Cells expressing CD11c are not required for efficient bacterial colonization of CLN. Chimeras of lethally irradiated wild type mice reconstituted with bone marrow from mice expressing the diphtheria toxin receptor behind a CD11c promoter were treated with diphtheria toxin or PBS (control). They were infected with *B*. *melitensis* with 10^9^ bacteria per mouse by the oral route. At 8 days post-infection, mice were sacrificed and organs analyzed for their bacterial loads. Data represent mean CFU per organ and SEM of results from two independent experiments with 4 and 5 mice per group, respectively.
(TIF)

S6 Fig Cells expressing Langerin are not required for efficient bacterial colonization of CLN. Mice expressing the diphtheria toxin behind a Langerin promoter were treated with diphtheria toxin or left untreated (control). They were infected with *B*. *melitensis* with 10^9^ bacteria per mouse by the oral route. At 8 days post-infection, mice were sacrificed and organs analyzed for their bacterial loads. Data represent mean CFU per organ and SEM of results from two independent experiments with 3 mice per group.
(TIF)

S7 Fig During gavage infection with *S*. Typhimurium, bacteria colonize CLN at higher densities than the spleen at early time points. C57BL/6 mice were infected with 10^5^ *S*. Typhimurium by gavage. At 2 or 3 days post-infection, mice were sacrificed and organs weighed and analyzed for their bacterial loads per gram tissue by plating homogenates on nutrient agar. Data represent mean and SEM of the pooled results from three (day 2) or two (day 3) independent experiments with 4 mice per group. * p ≤ 0.05.
(TIF)

S8 Fig Oral infection with *B*. *melitensis* induces genes in CLN favoring polarization towards or indicating presence of classically activated macrophages. C57BL/6 mice were infected with 10^9^ *B*. *melitensis* per mouse or mock infected by the oral route. At 15 days post-infection, mice were sacrificed, total RNA of the CLN was extracted and analyzed for expression of genes involved in inflammatory responses by reverse transcription real-time PCR. Results are given as fold expression versus the signal obtained for mock-infected mice. Data represent mean and standard deviations of one experiment with 5 mice per group. * p ≤ 0.05 as compared to mock infected expression levels.
(TIF)

S9 Fig Oral infection with *B*. *melitensis* induces only minor changes in expression of IL-10, TNFα, osteopontin and IL-18 in the CLN. C57BL/6 mice were infected with 10^9^ *B*. *melitensis* or mock infected by the oral route. At 5 h, 2, 8, 15 or 29 days post-infection, mice were sacrificed, total RNA of the CLN was extracted and analyzed for expression of genes involved in inflammatory responses by reverse transcription real-time PCR. Results are given as fold expression versus the signal obtained for mock-infected mice. Data represent means and standard deviations of two pooled independent experiments. * p ≤ 0.05 as compared to mock infected expression levels.
(TIF)

S10 Fig *B*. *melitensis* oral infection results in increase in absolute numbers but only minor changes in relative proportions or surface marker expression of CLN B and T lymphocyte populations. CLN from mice orally infected with 10^9^ *B*. *melitensis* for 15 days or mock-infected controls were prepared for flow cytometry. **(A)** Percentages of B and T lymphocytes of CLN from uninfected and *Brucella*-infected mice. **(B)** Different subpopulations of CD5^+^ T lymphocytes (CD8^+^ and CD4^+^, respectively) are shown with respect to their expression of CD62L and CD44. **(C)** and **(D)** B (CD19+) and CD5^+^ T lymphocytes were analyzed for their median fluorescence of (C) CD62L or (D) CD44. **(E)** Absolute numbers of B and T lymphocytes of CLN from uninfected and *Brucella*-infected mice. Data represent mean and SEM of pooled results from two independent experiments with a total of 8 (mock-infected) and 9 (infected) mice per group. * p ≤ 0.05 as compared to respective mock-infected control.
(TIF)

S11 Fig Even late stage CLN granulomas show no or little necrotic debris. Thin sections of cervical lymph nodes from mice orally infected with 10^9^ *B*. *melitensis* per mouse for (A) 5 h and (B) 50 days were stained with eosin-hematoxylin. Whereas no obvious changes can be observed at 5 h, compact, epitheloid granulomas without or only little necrosis can be observed at day 50 (white arrow heads).
(TIF)

S12 Fig Base level expression of genes involved in regulation of inflammatory processes varies in lymph nodes of different locations. Untreated C57BL/6 mice were sacrificed, total RNA of different lymph nodes was extracted and analyzed for expression of genes involved in inflammatory responses by reverse transcription real-time PCR. Results are given as fold expression compared to the signal obtained for CLN. Data represent means and standard deviations of two pooled independent experiments with 4 and 5 mice. * p ≤ 0.05 as compared to CLN expression levels.
(TIF)
