# Supplementary material for: Cervical Lymph Nodes as a Selective Niche for Brucella during Oral Infections
Source: PLoS One. 2015 Apr 28;10(4):e0121790. doi: 10.1371/journal.pone.0121790 (PMC4412401; doi:10.1371/journal.pone.0121790)
Supplement: S1 Table — (DOC) [file pone.0121790.s001.doc]

**Table S1: Primers used for analysis of gene expression upon oral infection.** All sequences are given in 5’ 🡪 3’ orientation.

| **Gene** | **Forward primer** | **Reverse primer** |
| --- | --- | --- |
| HPRT | AGCCCTCTGTGTGCTCAAGG | CTGATAAAATCTACAGTCATAGGAATGGA |
| IL-6 | GAGGATACCACTCCC AACAGACC | AAGTGCATCATCGTTGTTCATACA |
| IL-10 | GGT TGCCAAGCCTTATCGGA | ACCTGCTCCACTGCCTTGCT |
| IFNγ | TCAAGTGGCATAGATGTGGAAGAA | TGGCTCTGCAGGATTTTCATG |
| GranB | ATCAAGGATCAGCAGCCTGA | CATGATGTCATTGGAGAATGTCT |
| FoxP3 | AGGAGCCGCAAGCTAAAAGC | TGCCTTCGTGCCCACTGT |
| TNFα | CATCTTCTCAAAATTCGAGTGACAA | TGGGAGTAGACAAGGTACAACCC |
| NOS2 | CAGCTGGGCTGTACAAACCTT | CATTGGAAGTGAAGCGTTTCG |
| IL-12b | AAATTACTCCGGACGGTTCA | ACAGAGACGCCATTCCACAT |
| IL-18 | TGTCTACCCTCTCCTGTAAGAACA | CTGGAACACGTTTCTGAAAGAAT |
| CCL2 | GCCTGCTGTTCACAGTTGC | ATTGGGATCATCTTGCTGGT |
| CCR2 | GAAGAGGGCATTGGATTCAC | GTATGCCGTGGATGAACTGA |
| Arg1 | GGCAGAGGTCCAGAAGAATG | AGCATCCACCCAAATGACAC |
| Ptgs2/Cox2 | ACCTCTGCGATGCTCTTCC | TCATACATTCCCCACGGTTT |
| IDO-1 | CCCTGGGGTACATCACCAT | GAGAGCTCGCAGTAGGGAAC |
| IL-23a | CAGCAGCTCTCTCGGAATCT | ACTGGATACGGGGCACATTA |
| MAFB | TATTCCAAGGAGTCGCCAAG | TTCCTGGGACGAACTGAGTC |
| Klf-4 | GCCACCCACACTTGTGACTAT | CGTCCCAGTCACAGTGGTAA |
| IL-4 | ACTCTTTCGGGCTTTTCGAT | TTGCATGATGCTCTTTAGGC |
