# Supplementary figures and images for: Cervical Lymph Nodes as a Selective Niche for Brucella during Oral Infections
Source: PLoS One. 2015 Apr 28;10(4):e0121790. doi: 10.1371/journal.pone.0121790 (PMC4412401; doi:10.1371/journal.pone.0121790)

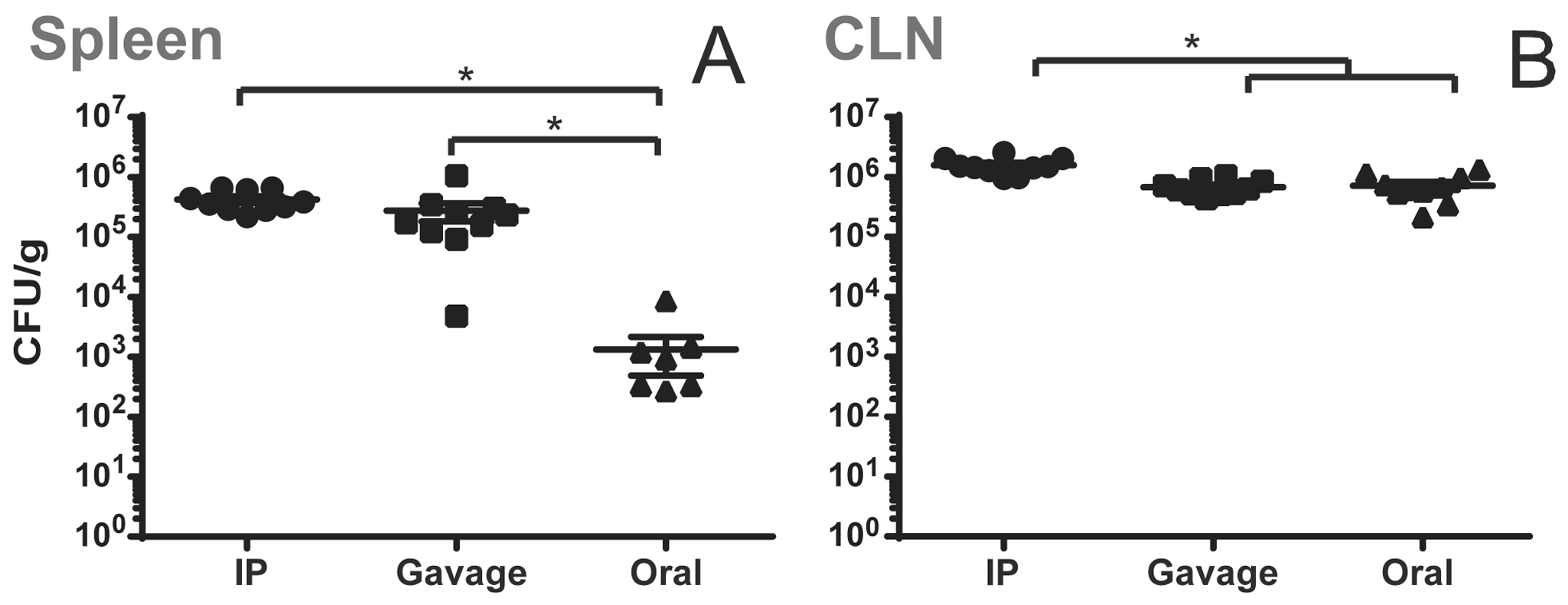

Supplement: S1 Fig — C57BL/6 mice were infected by intraperitoneal injection (106 bacteria/mouse), intragastric by gavage or by the oral route (both at 109 bacteria/mouse). At 8 days post-infection, mice were sacrificed and organs weighed and analyzed for their bacterial loads by plating homogenates on nutrient agar. Data represent mean of colony-forming units per gram tissue and SEM of the pooled results from two independent experiments with 5 mice per group. Non-infected organs are not shown due to logarithmic scale. * p ≤ 0.05. (TIF) [file pone.0121790.s002.tif]

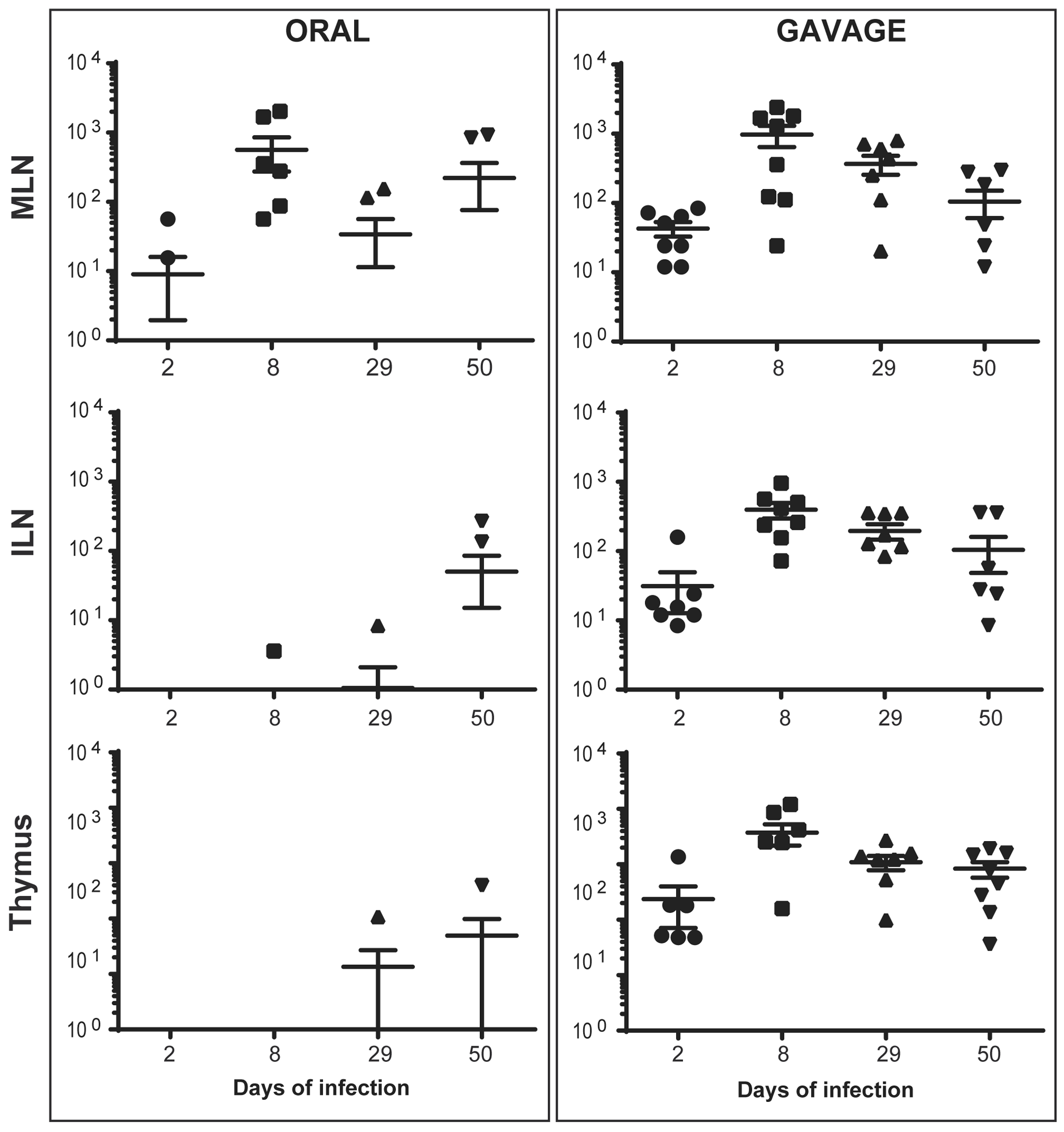

Supplement: S2 Fig — C57BL/6 mice were infected with B. melitensis with 109 bacteria per mouse by gavage or by the oral route. At 2, 8, 29 or 50 days post-infection, mice were sacrificed and organs analyzed for their bacterial loads by plating homogenates on nutrient agar. Data represent mean CFU per organ and SEM of the pooled results from two independent experiments with 4 mice per group. (TIF) [file pone.0121790.s003.tif]

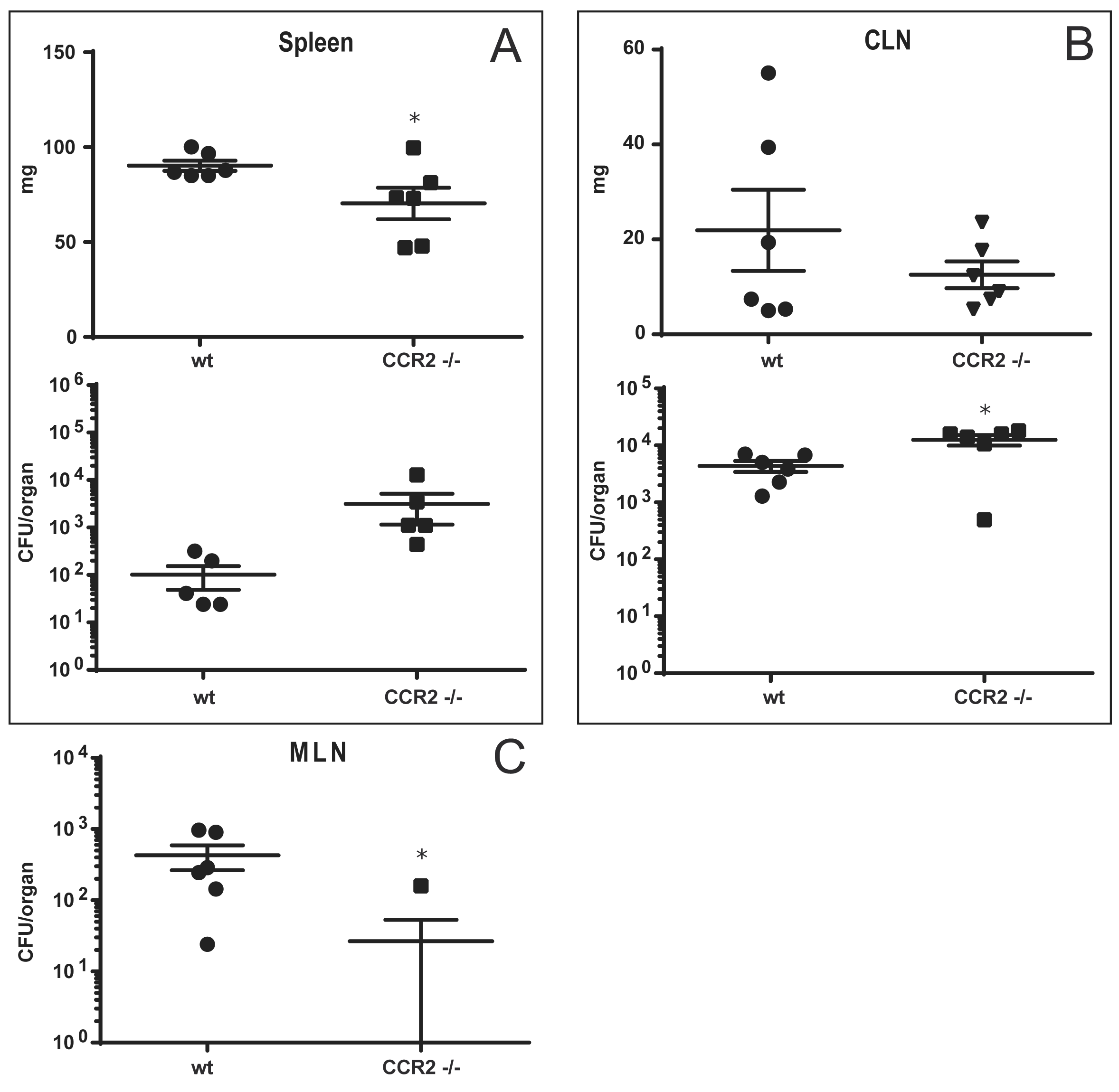

Supplement: S3 Fig — CCR2 deficient mice or wild type mice were infected with B. melitensis with 109 bacteria per mouse by the oral route. At 8 days post-infection, mice were sacrificed and organs analyzed for their bacterial loads. Data represent mean CFU per organ and SEM of results from two independent experiments with three mice per group. (TIF) [file pone.0121790.s004.tif]

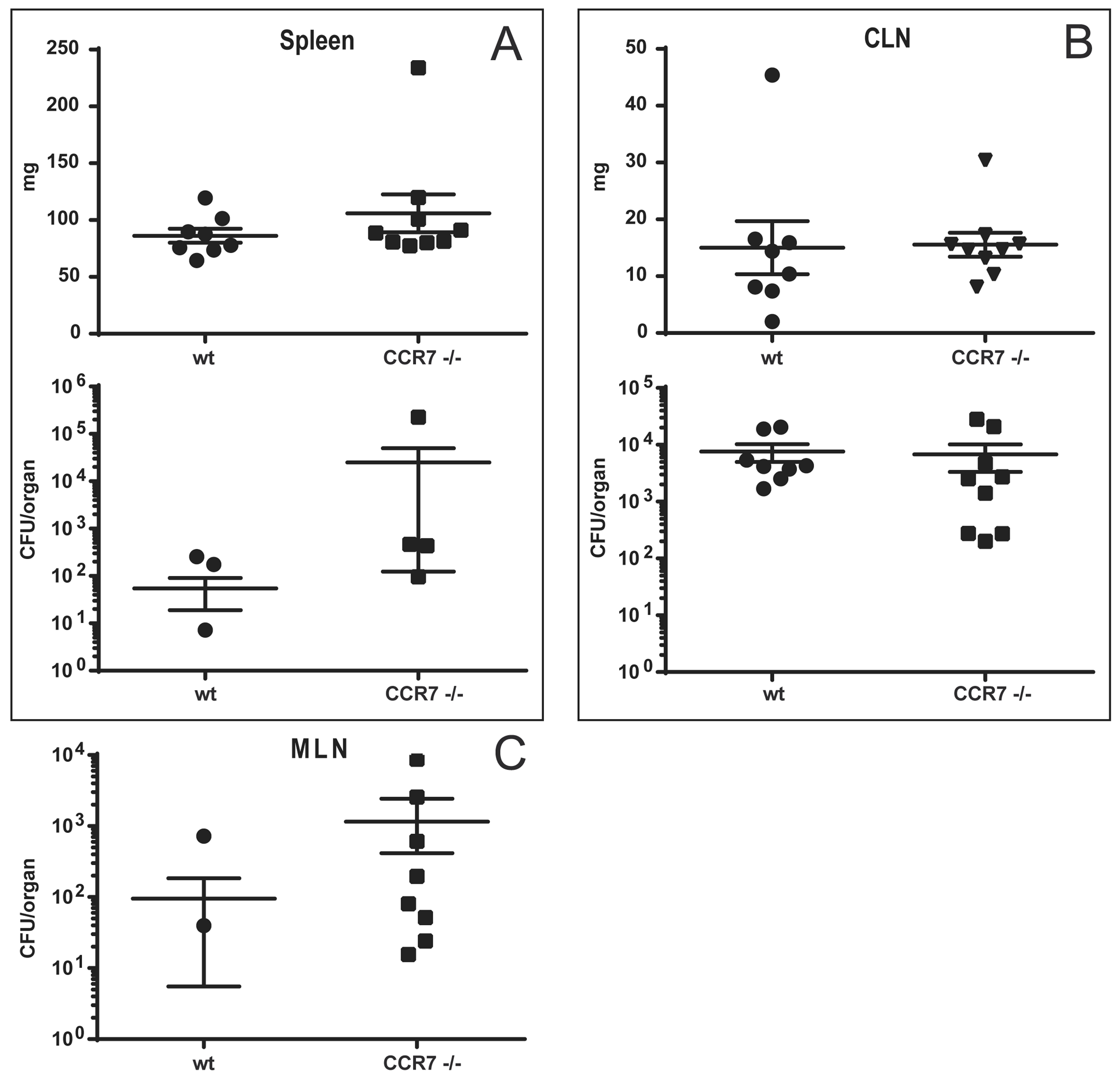

Supplement: S4 Fig — Mice with a CCR7 deficiency or wild type mice were infected with B. melitensis with 109 bacteria per mouse by the oral route. At 8 days post-infection, mice were sacrificed and organs analyzed for their bacterial loads. Data represent mean CFU per organ and SEM of results from two independent experiments with 3 mice per group. (TIF) [file pone.0121790.s005.tif]

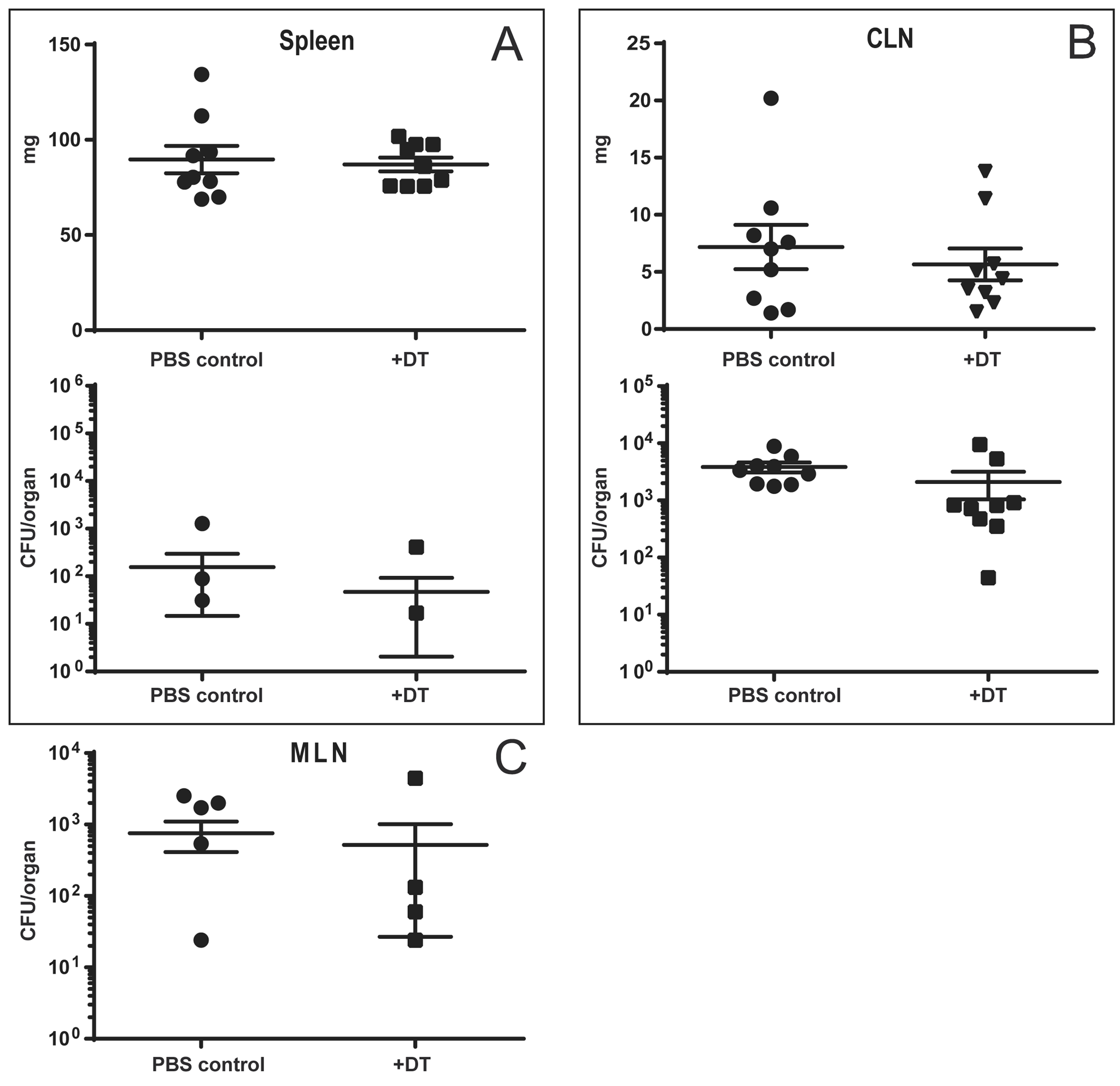

Supplement: S5 Fig — Chimeras of lethally irradiated wild type mice reconstituted with bone marrow from mice expressing the diphtheria toxin receptor behind a CD11c promoter were treated with diphtheria toxin or PBS (control). They were infected with B. melitensis with 109 bacteria per mouse by the oral route. At 8 days post-infection, mice were sacrificed and organs analyzed for their bacterial loads. Data represent mean CFU per organ and SEM of results from two independent experiments with 4 and 5 mice per group, respectively. (TIF) [file pone.0121790.s006.tif]

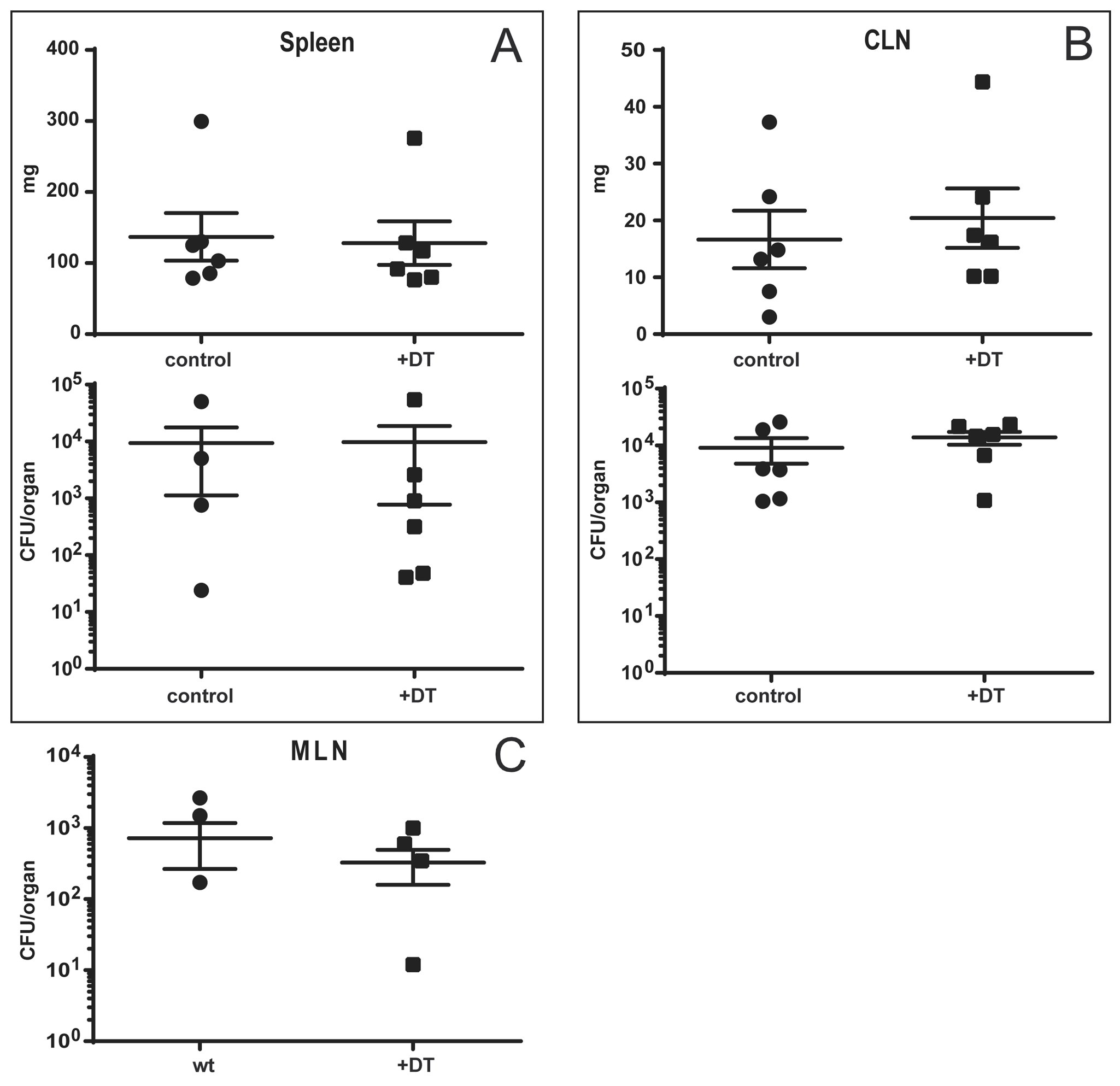

Supplement: S6 Fig — Mice expressing the diphtheria toxin behind a Langerin promoter were treated with diphtheria toxin or left untreated (control). They were infected with B. melitensis with 109 bacteria per mouse by the oral route. At 8 days post-infection, mice were sacrificed and organs analyzed for their bacterial loads. Data represent mean CFU per organ and SEM of results from two independent experiments with 3 mice per group. (TIF) [file pone.0121790.s007.tif]

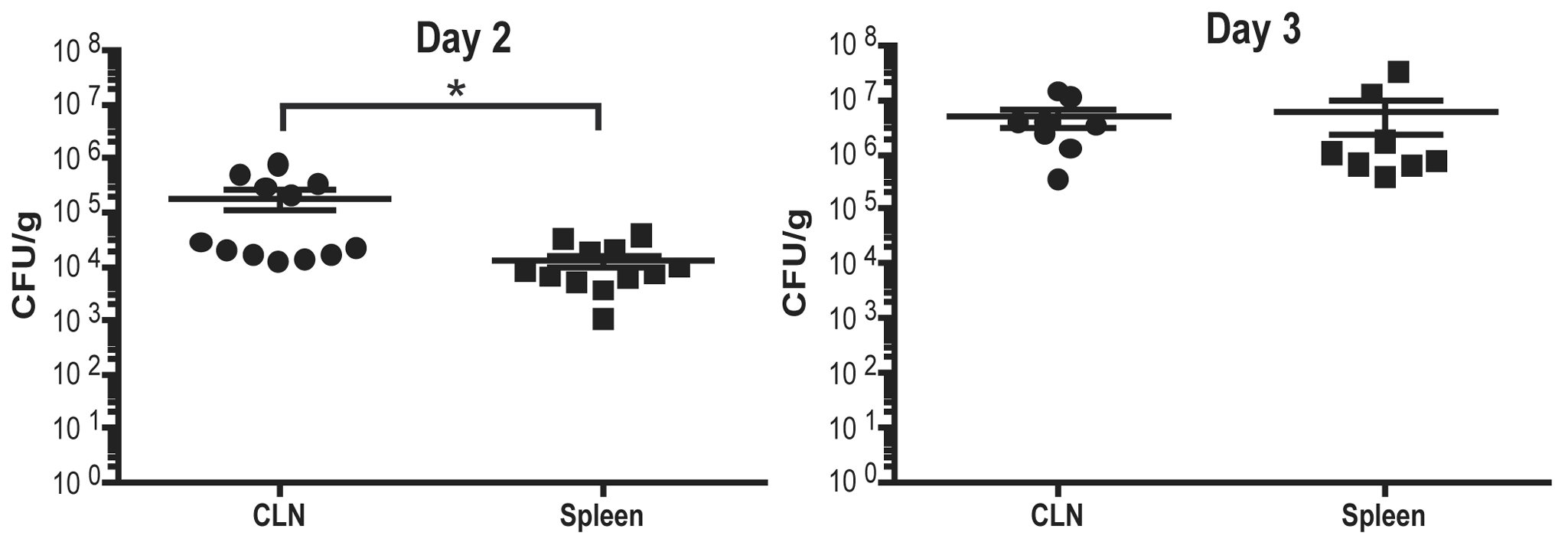

Supplement: S7 Fig — C57BL/6 mice were infected with 105 S. Typhimurium by gavage. At 2 or 3 days post-infection, mice were sacrificed and organs weighed and analyzed for their bacterial loads per gram tissue by plating homogenates on nutrient agar. Data represent mean and SEM of the pooled results from three (day 2) or two (day 3) independent experiments with 4 mice per group. * p ≤ 0.05. (TIF) [file pone.0121790.s008.tif]

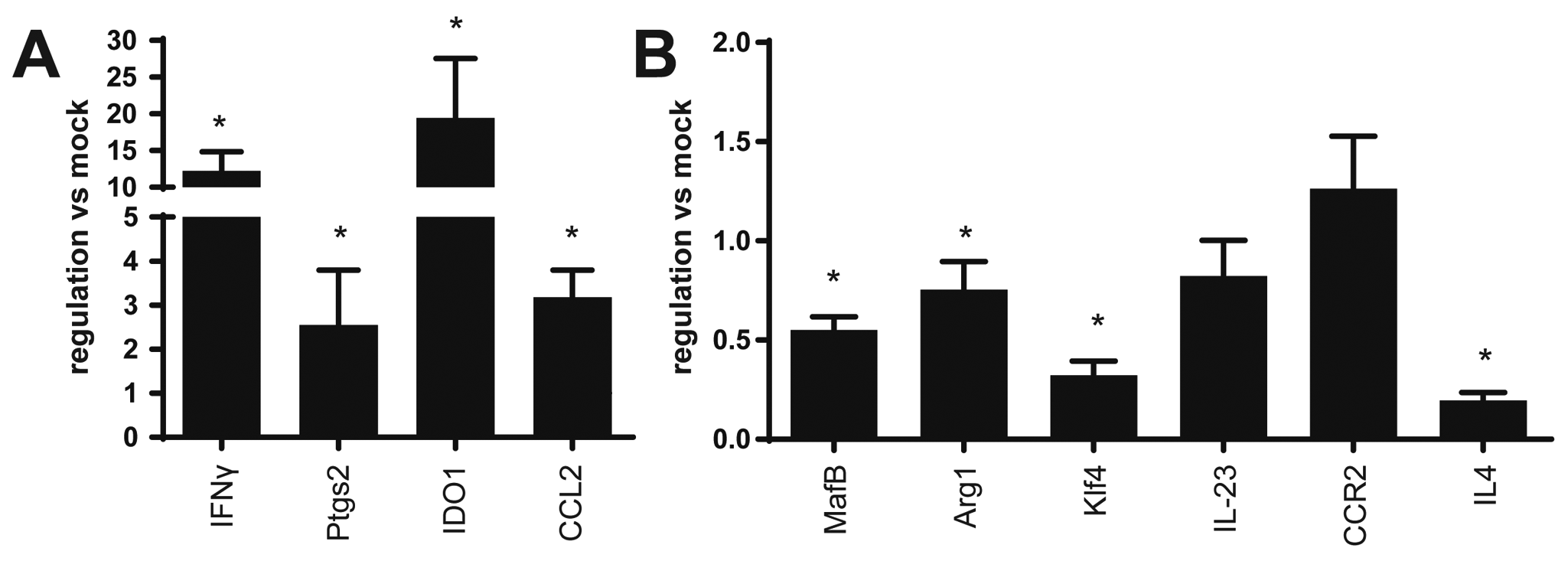

Supplement: S8 Fig — C57BL/6 mice were infected with 109 B. melitensis per mouse or mock infected by the oral route. At 15 days post-infection, mice were sacrificed, total RNA of the CLN was extracted and analyzed for expression of genes involved in inflammatory responses by reverse transcription real-time PCR. Results are given as fold expression versus the signal obtained for mock-infected mice. Data represent mean and standard deviations of one experiment with 5 mice per group. * p ≤ 0.05 as compared to mock infected expression levels. (TIF) [file pone.0121790.s009.tif]

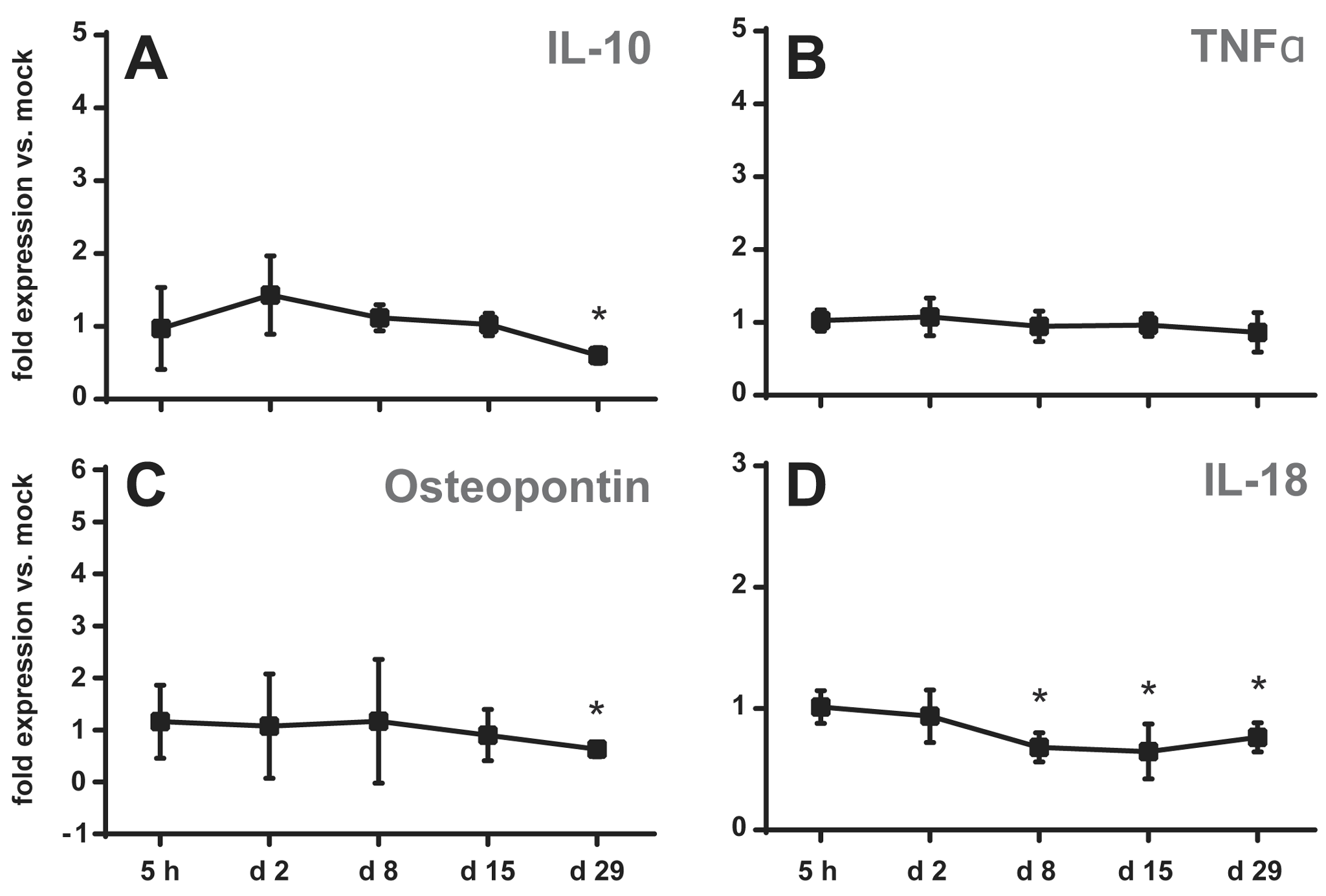

Supplement: S9 Fig — C57BL/6 mice were infected with 109 B. melitensis or mock infected by the oral route. At 5 h, 2, 8, 15 or 29 days post-infection, mice were sacrificed, total RNA of the CLN was extracted and analyzed for expression of genes involved in inflammatory responses by reverse transcription real-time PCR. Results are given as fold expression versus the signal obtained for mock-infected mice. Data represent means and standard deviations of two pooled independent experiments. * p ≤ 0.05 as compared to mock infected expression levels. (TIF) [file pone.0121790.s010.tif]

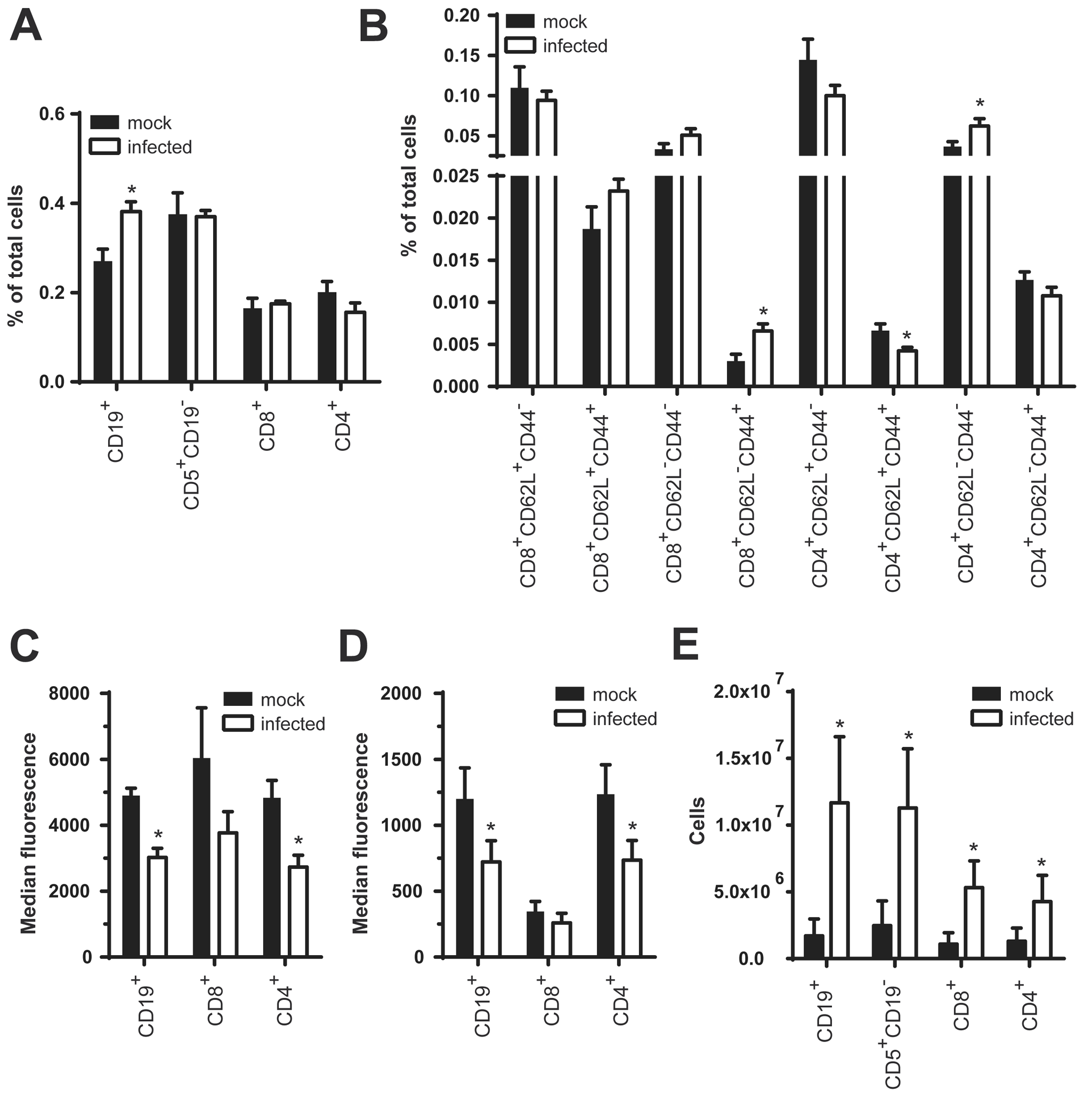

Supplement: S10 Fig — CLN from mice orally infected with 109 B. melitensis for 15 days or mock-infected controls were prepared for flow cytometry. (A) Percentages of B and T lymphocytes of CLN from uninfected and Brucella-infected mice. (B) Different subpopulations of CD5+ T lymphocytes (CD8+ and CD4+, respectively) are shown with respect to their expression of CD62L and CD44. (C) and (D) B (CD19+) and CD5+ T lymphocytes were analyzed for their median fluorescence of (C) CD62L or (D) CD44. (E) Absolute numbers of B and T lymphocytes of CLN from uninfected and Brucella-infected mice. Data represent mean and SEM of pooled results from two independent experiments with a total of 8 (mock-infected) and 9 (infected) mice per group. * p ≤ 0.05 as compared to respective mock-infected control. (TIF) [file pone.0121790.s011.tif]

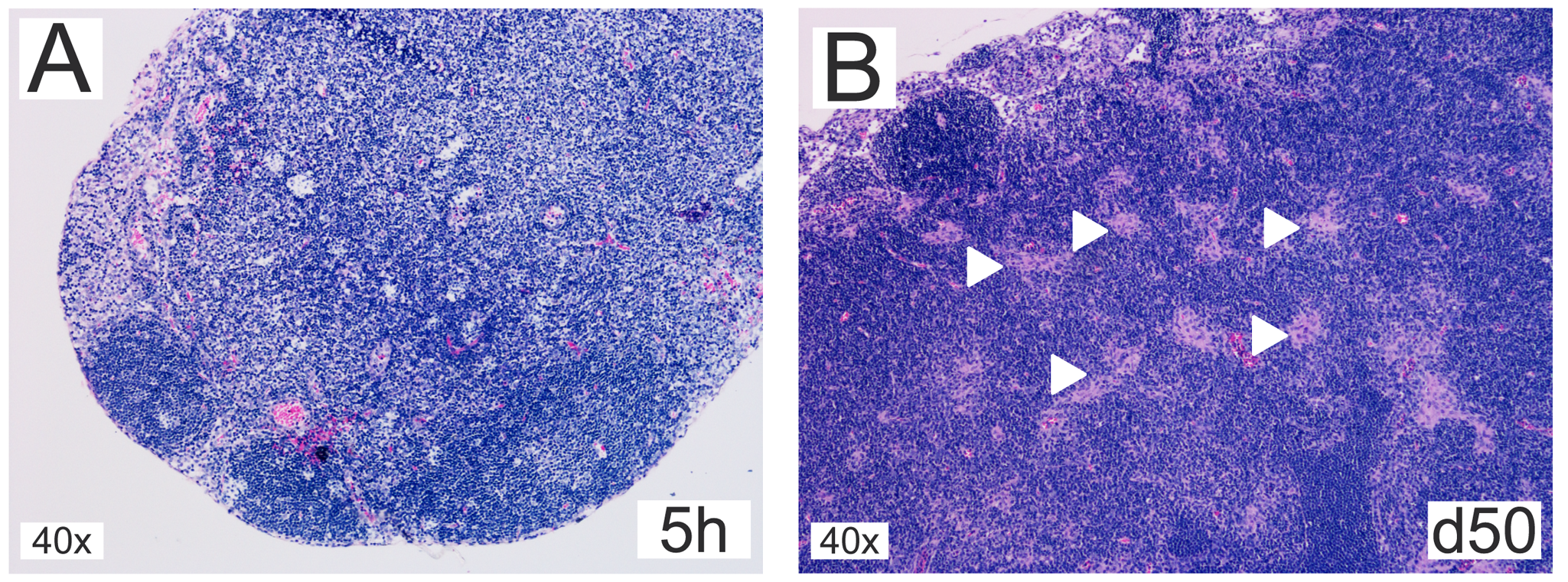

Supplement: S11 Fig — Thin sections of cervical lymph nodes from mice orally infected with 109 B. melitensis per mouse for (A) 5 h and (B) 50 days were stained with eosin-hematoxylin. Whereas no obvious changes can be observed at 5 h, compact, epitheloid granulomas without or only little necrosis can be observed at day 50 (white arrow heads). (TIF) [file pone.0121790.s012.tif]

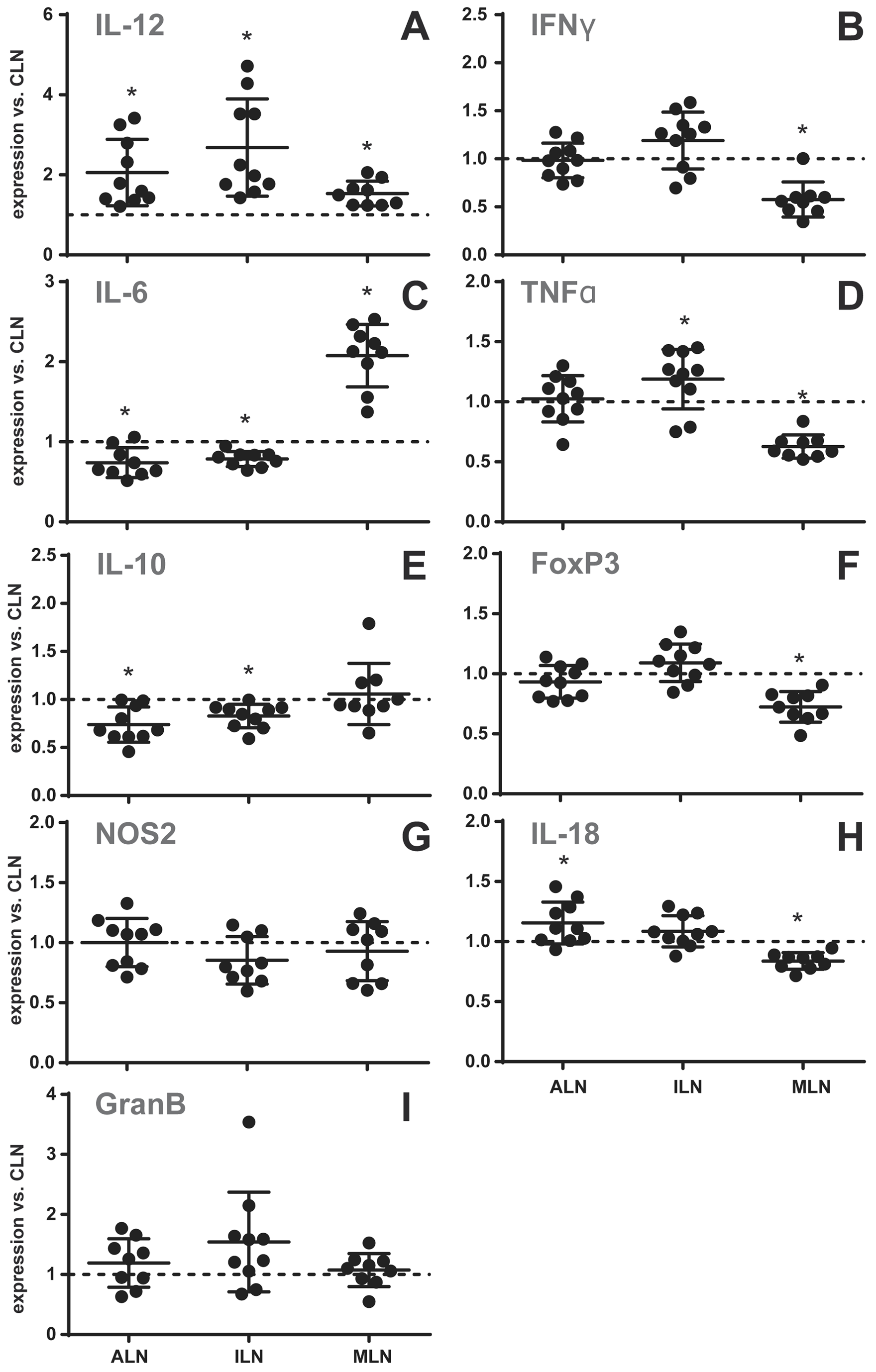

Supplement: S12 Fig — Untreated C57BL/6 mice were sacrificed, total RNA of different lymph nodes was extracted and analyzed for expression of genes involved in inflammatory responses by reverse transcription real-time PCR. Results are given as fold expression compared to the signal obtained for CLN. Data represent means and standard deviations of two pooled independent experiments with 4 and 5 mice. * p ≤ 0.05 as compared to CLN expression levels. (TIF) [file pone.0121790.s013.tif]
